# The Carabidae (Coleoptera) of Shada Al-A’Ala Nature Reserve, Southwestern Saudi Arabia, with description of a new species of Paussinae

**DOI:** 10.3897/zookeys.812.30937

**Published:** 2019-01-03

**Authors:** Mahmoud S. Abdel-Dayem, Ali A. Elgharbawy, Iftekhar Rasool, Peter Nagel, Hathal M. Aldhafer

**Affiliations:** 1 King Saud University Museum of Arthropods (KSMA), Plant Protection Department, College of Food and Agriculture Sciences, King Saud University, P.O. Box 2460 Riyadh 11451, Saudi Arabia King Saud University Museum of Arthropods Riyadh Saudi Arabia; 2 Entomology Department, Faculty of Science, Cairo University, Giza 12613, Egypt Cairo University Giza Egypt; 3 Zoology Department, Faculty of Science, Al Azhar University, Nasr City, Cairo, Egypt Al Azhar University Cairo Egypt; 4 Biogeography, Department of Environmental Sciences, Faculty of Science, University of Basel, St. Johanns-Vorstadt 10, 4056 Basel, Switzerland University of Basel Basel Switzerland

**Keywords:** Baha, ground beetles, Shada, endemics, faunistic inventory, new records, new species, nature reserve, Saudi Arabia, zoogeography

## Abstract

We report the Carabidae collected at the Shada Al-A’Ala Nature Reserve (SANR) in Baha Province in southwestern Saudi Arabia during 2013–2015. In total, 62 carabid species and subspecies representing 39 genera, 17 tribes, and 10 subfamilies were identified, including one new species, *Paussusminutulus* Nagel & Rasool, **sp. n**, four new country records, and 24 species that are new provincial records for Baha. The carabid fauna was dominated by the Lebiini with 19 species. A high number of species were rarely collected (34 species) in comparison to the more abundant and common species (9 species). The highest number of species (52 species) was collected during autumn. The carabids of SANR are represented by a large component of Afrotropical faunal elements (28.1%) and smaller numbers of Oriental species (3.5%) and endemic taxa (5.3%). In comparison to Garf Raydah Nature Reserve in Asir Province, also in southwestern Saudi Arabia, SANR had an equal number of carabids sharing 64.5% of the species but with lower number of endemic elements. Our study can serve as a component for implementing a conservation plan for SANR using carabid beetles as sentinel taxa. These research results may support future ecological studies on SNAR carabids.

## Introduction

Over the past three decades, numerous new wildlife protected areas have been established in Saudi Arabia (SA). Not only the number of national parks has increased but also newly established nature reserves, wildlife sanctuaries, and other protected landscapes and biosphere reserves (Abuzinada 2003). It is noted that SA has currently 16 protected areas and 12 national parks (Abuzinada 2003; SWA 2018). For this network of protected areas, biodiversity monitoring is fundamental for effective management. The invertebrate fauna of these protected areas has attracted relatively little attention as compared to those of vertebrates (Abuzinada et al. 2005), although international conventions signed by SA as a member of the Convention on Biological Diversity since 2001 (CBD 2011). Recent insect biodiversity survey and monitoring research projects in several protected areas in SA have been conducted by King Saud University Museum of Arthropods, Riyadh; resulting in several faunistic and ecological works being published (Aldhafer et al. 2012, 2016; Sharaf et al. 2013; Abdel-Dayem et al. 2016, 2017, 2018; El-Hawagry et al. 2016, 2017, 2018). One of these recent projects was focused on the insect biodiversity of Shada Al-A’Ala Nature Reserve (SANR) in Baha Province in southwestern SA. The location and elevation range (470–2,222 m) of the SANR provides relative high rainfall, diverse microclimates, and a distinct biodiversity (SWA 2018). The SANR is undoubtedly one of the most interesting protected areas in SA because of the existence of unique treasure trove of biological diversity. About 22% (495 plant species) of the total SA flora has been reported from the Shada Mountains including 19 endemic plant species and 43% endangered species (Thomas et al. 2017). The SANR harbours important faunas, including griffon vultures and other endemic birds of the southwestern mountains and carnivores (e.g. the rock fox, caracal, striped hyena, wolf, genet, and the Arabian leopard) (SWA 2018). Regarding insects, 119 species of flies (Diptera) have been reported from the SANR (El-Hawagry et al. 2016).

The Carabidae have a cosmopolitan distribution and form one of the most diverse and abundant families of insects constituting a considerable component of the soil fauna (Duchesne et al. 1999). These beetles play an important role in ecosystems as polyphagous predators, whereas others are phytophagous (Thiele 1977). Additionally, carabid beetles play significant role as bioindicators in habitat management, landscape ecology, conservation, pollution, climatic changes and soil characteristics (Rainio and Niemelä 2003; Avgın and Luff 2010; Koivula 2011; Kotze et al. 2011). There are currently 33,920 valid species (Lorenz 2005), 183 of which have been reported from SA (Abdel-Dayem et al. 2018). Unexpectedly, the number of previously published carabid species records from Baha Province are low (30 species) (Mateu 1986; Balkenohl 1994; El-Hawagry et al. 2013; Moore and Robertson 2014; Häckel and Azadbakhsh 2016; Rasool et al. 2017, 2018a, b). In the most recent study of the Baha insect fauna (El-Hawagry et al. 2013), 17 carabid species were reported, with only ant nest beetle, *Paussuscephalotes* Raffray, 1886 known from SNAR. Rasool et al. (2017, 2018a, b) have reviewed the subtribes Cymindidina, Dromiusina and Lebiina of tribe Lebiini from southwestern SA and described *Lebiaraeesae* Rasool, Abdel-Dayem & Felix, 2018 and reported *Calodromiusmayeti* (Bedel, 1907), *Dromiusbuettikeri* Mateu, 1990, *Lebiaauberti* Fairmaire, 1892, *L.nilotica* Chaudoir, 1871, *Matabelearabica* Mateu, 1986, *Mesolestesquadriguttatus* Mateu, 1979, *Metadromiusarabicus* Mateu, 1979, *Met.brittoni* (Basilewsky, 1948), *Microlestesdiscoidalis* (Fairmaire, 1892), *Mic.infuscatusfragilis* Mateu, 1956 and *Zolotarevskyellarhytidera* (Chaudoir, 1876) from SANR.

However, despite the urgent conservation concerns associated with the SANR reserve, there have been no studies focused on beetles including the Carabidae. Thus, the objectives of this study are to provide a thorough baseline inventory of the carabid fauna of the SANR and to analyze its zoogeographical affinities. This information will assist in providing an essential cognitive basis for future management of this reserve. Additionally, our results will allow for future ecological studies of carabids of SNAR and will contribute to the overall knowledge of Carabidae of SA, the largest country of the Arabian Peninsula.

## Methods

### Study area

**Figure 1.**
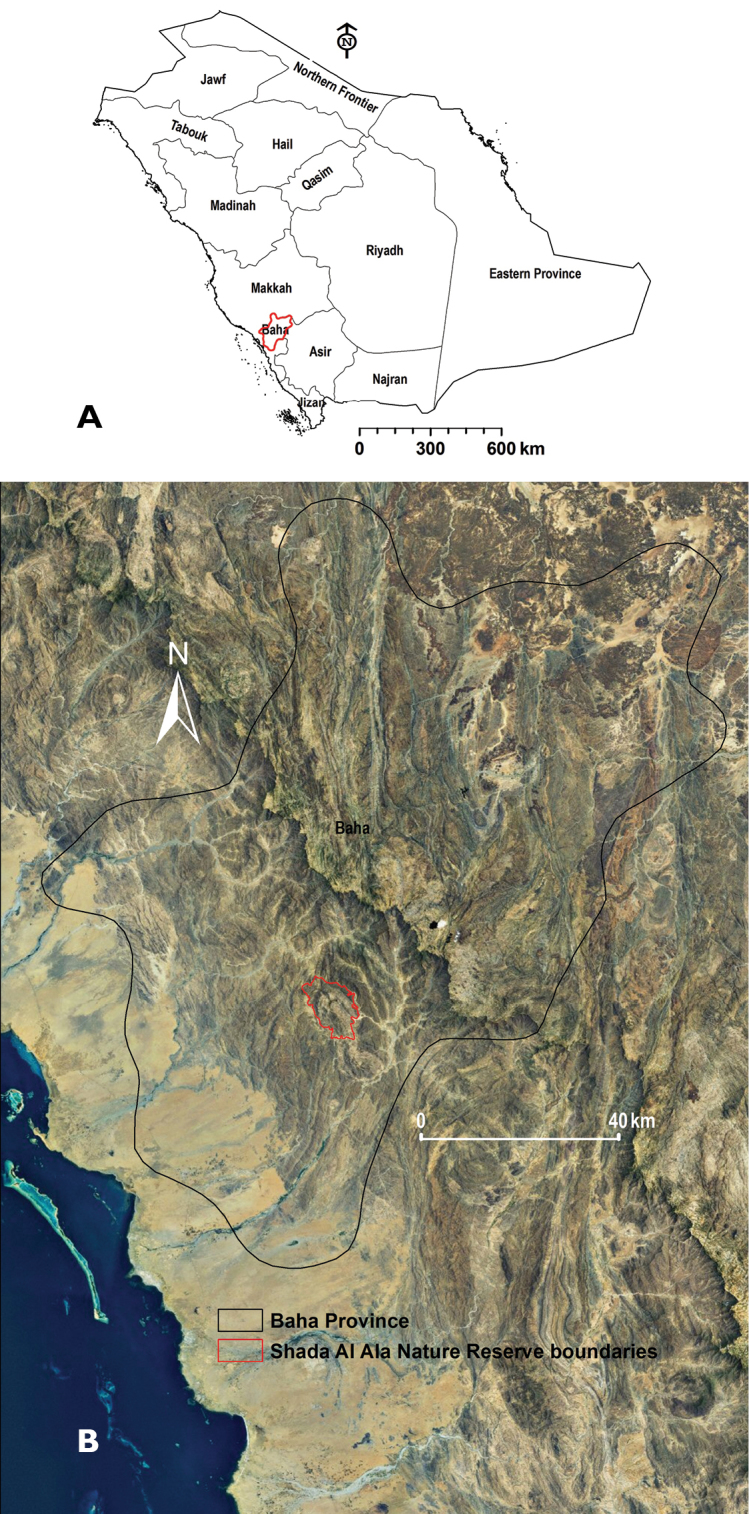
**A** Map of Saudi Arabia showing location of Baha Province **B** Location of Shada Al-A’Ala Nature Reserve within Baha Province.

In Shada Al-A’Ala (Upper Shada) Mountain, an outlier of the Sarawat Mountains to the west, the SANR was established in 2002. The reserve is located (latitudes 19°48.894'–19°52.578'N and longitudes 41°17.130'–41°21.000'E) in Al-Mekhwa District (Baha Province); about 20 km southwest of Al-Mekhwa City, the capital of the district (Fig. 1). The SANR occupies an area of 67 km^2^ and rises about 2,222 m. There is a perennial small freshwater stream in Wadi Neera at the west and southwest part of the reserve. Geologically the area belongs to the greater Afro-Arabian shield, which is a part of the Precambrian crust plate and is generally exposed and locally covered by tertiary volcanic rocks (Schmidt et al. 1972). There are terraced fields used by small local communities; these fields are very small scale and are used to grow distinctive varieties of coffee, banana, lemon, and natural figs (SWA 2018). The climate is similar to the uplands of southwestern SA. It is highly variable and characterized by cool winters, warmer partly cloudy summer, and high rainfall. The average annual temperature of 26.2 °C, and average annual rainfall of about 200 mm, and with wettest period concentrated between March and May (43% annual precipitation) (El-Hawagry et al. 2016).

The vegetation is rich, with the Leguminosae (Fabaceae) and composites (Asteraceae) having the highest contribution, followed by graminoides (Poaceae) (Al Zubaide et al. 2017, Thomas et al. 2017). The vegetation comprises 72.4% perennials and 27.6% annuals; represented by 17.2% trees, 51.8% shrubs, and 31.1% weeds (Al Zubaide et al. 2017). The vegetation at the foothills of Shada Al-A’Ala Mountain consists predominantly of subtropical *Acacia* thorn woodlands extending from the base up to 1500 m a.s.l. The vegetation above 1000 m elevation is dominated by *Acaciaasak* (Thomas et al. 2017). Higher up, above the *Acacia* zone, there are shrubs of Barbary fig or cactus pear, *Opuntiaficus*-*indica* (L.) Mill. (Cactaceae). For more details on the vegetation in SANR (see El-Hawagry et al. 2016).

### Beetle collection

As part of a research project for studying the insect biodiversity in the SANR the adult ground beetles were sampled from 2013–2015. The sampling was conducted at various sites in varied habitats at 13 different elevation levels (Table 1) within the SANR. The geographical coordinate data of each collecting location were recorded using GPS Garmin, Montana 650 unit (Garmin Instruments Inc., Olathe, Kansas, USA).

**Table 1.** List of collecting elevation levels and geographical coordinates, in Shada Al-A’Ala Nature Reserve, southwestern SA.

No.	Elevation (m)	Latitude (N)	Longitude (E)
1	471	19°44.870', 41°20.008'	
2	825	19°52.717', 41°18.712'	
3	851	19°52.685', 41°18.663'	
4	892	19°52.598', 41°18.672'	
5	1.008	19°52.023', 41°18.157'	
6	1.225	19°51.762', 41°18.089'	
7	1.325	19°51.066', 42°18.037'	
8	1.388	19°51.387', 41°18.187'	
9	1.448	19°47.511', 41°18.258'	
10	1.474	19°50.710', 41°18.267'	
11	1.563	19°50.329', 41°18.604'	
12	1.611	19°50.411', 41°18.686'	
13	1.666	19°50.575', 41°18.691'	

Collected beetles were initially sorted to morphospecies level, mounted and then identified to species levels. Some species were sent to experts for identification or confirmation, as indicated in the remarks. The specimens are deposited in the collection of King Saud University Museum of Arthropods (KSMA), King Saud University, Riyadh, SA.

The description of the new species of *Paussus* was assisted using a Leica M205C dissecting microscope with 10× eyepieces and Planapo 1.0× and 1.6× front lenses, allowing magnification up to 240×. An eyepiece micrometer was used for measurements.

### Classification and nomenclature

The subfamily and tribal classification of the family and nomenclature of the species in this study follows the Catalogue of Palaearctic Coleoptera (Löbl and Löbl 2017). However, the taxonomic order of species in the genus *Sphaerotachys* J. Miller, 1926 (Trechinae, Bembidiini) follows Sciaky and Vigna Taglianti (2003). The subfamilies, tribes, genera and species are listed alphabetically.

### Faunal list

For each species, the following information is provided: current nomenclatural combinations, material examined, zoogeography, distribution, published records in SA, and remarks. The label data for examined specimens are listed as follows: elevation level within the SANR, followed by the date of collection (months as Roman numerals), the collecting method (handpicking (HP), light traps (LT), malaise traps (MT), pitfall traps (PT), sweeping net (SW) and vacuuming (VC)) and the number of examined specimens followed by sex (♂ for male, ♀ for female, ex(s) for example with unidentified sex). The material examined is arranged in ascending order with respect to the elevation, then chronologically with respect to the month of collection. A semicolon separates different records; if these are from the same elevation, the elevation is listed only at the beginning with the older record.

### General distribution and zoogeography

The zoogeography, which were used in the analysis of carabid faunal affinity, were assigned for each species using the zoogeographic realms of the world suggested by Holt et al. (2013). The zoogeography is based on their modern general geographical distributions (each country is represented by two capital letters according to ISO 3166 “ISO Alpha VC–2 Country code”: The Nations Online Project: https://www.nationsonline.org/oneworld/country_code_list.htm :) provided by Löbl and Löbl (2017) and Lorenz (2017) unless otherwise stated.

## Results

During this study, 3,287 adult carabid beetles were collected from SANR, comprising 62 species from 39 genera within 17 tribes and 10 subfamilies. These species include the description of a new species (*Paussusminutulus* sp. n.), three SA endemic species and six confined to Arabian Peninsula. Four species have not been previously recorded from SA, and 24 species recorded for the first time from Baha Province. The details of these species are provided in the faunal list below.

**Figure 2.**
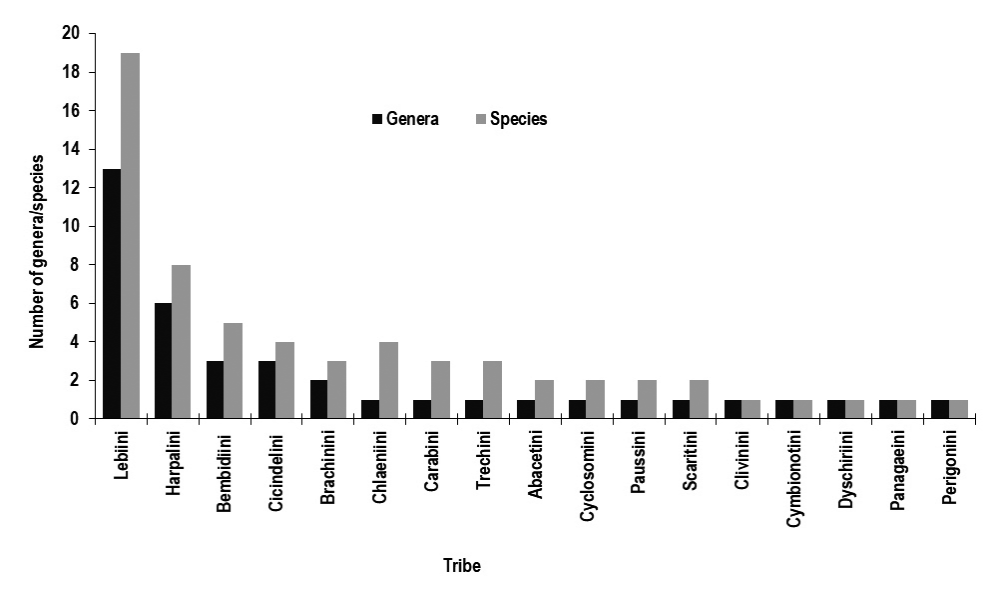
The number of genera and species for each tribe of ground beetles recorded between 2013–2015, from Shada Al-A’Ala Nature Reserve, southwestern Saudi Arabia.

Of the carabids collected from SNAR, the most diverse tribe was the Lebiini, represented by 19 species (30.6% of the total species) in 13 genera (33.3% of the total genera) (Fig. 2). About 50% of the tribes are represented by one or two species. Nine species (14.5%) are classified as abundant and common species; *Lebianilotica*, *Metadromiusarabicus*, *Sphaerotachysconspicuus* (Schaum, 1863) were the most abundant species, comprising 50.4% of the total catch. Twenty species (32.3%) are considered rare, represented by four or fewer individuals collected over the two years. The maximum number of species were collected during autumn (52 species). The genus *Anthracus* Motschulsky, 1850 was recorded for the first time for SA. Three species have been identified to the genus level, belonging to *Amblystomus*, *Metadromius* and *Singilis*.

### Faunal List

#### Brachininae

##### Brachinini

###### Brachinus crepitans

Taxon classificationAnimaliaColeopteraCarabidae

(Linnaeus, 1758)

####### Material examined.

471 m: 10.XII.2014, LT, 1♂, 1♀.

####### General distribution and zoogeography.

AL, DZ, AM, AT, AZ, BY, BE, BA, BG, HR, CY, CZ, DK, EE, FI, FR, GE, DE, GB, GR, HU, IR, IQ, IE, IT, JO (Nasir and Katbeh-Bader 2017), KZ, KG, LV, LB, LT, LU, MK, MD, ME, NL, NO, PL, PT, RO, RU, SA, SK, SI, ES, SE, CH, SY, TJ, TN, TR, TM, UA, UZ. This range exemplifies PAL_SAR.

####### Published records.

Asir (Abdel-Dayem et al., 2018), Riyadh (Abdel-Dayem et al. 2017). New provincial records for Baha.

####### Remarks.

A rare species. The adult beetles were collected during autumn by hand picking under stones along the edge of a freshwater stream flowing through *Acacia* thorn woodlands. Mahmoud Abdel-Dayem identified this species.

###### Brachinus dorsalis

Taxon classificationAnimaliaColeopteraCarabidae

Dejean, 1831

####### Material examined.

892 m: 14.II.2014, LT, 1♂; 02. III.2014, LT, 1♂; 18.X.2014, LT, 1♀; 14.XI.2015, LT, 1♂; 08.XII.2014, LT. 1♂; 09.XII.2014, LT, 1♂.

####### General distribution and zoogeography.

SA, YE. END_AR species.

####### Published records.

Asir (Abdel-Dayem et al. 2018), Jizan (Mateu 1990). New provincial records for Baha.

####### Remarks.

A rare species that was collected during autumn and winter by light trapping close to a freshwater stream flowing through *Acacia* thorn woodlands. Mahmoud Abdel-Dayem identified this species.

###### Pheropsophus africanus

Taxon classificationAnimaliaColeopteraCarabidae

(Dejean, 1825)

####### Material examined.

471 m: 03.III.2015, LT, 1♂; 15.XI.2015, LT, 1♂, 3♀; 10.XII.2014, HP, 9♂, 2♀.

####### General distribution and zoogeography.

AE, DZ, EG (including Sinai), ER, ET, IL, IQ, IR, JO (Nasir and Katbeh-Bader 2017), LY, MA, NE, SA, SD, TD, TN, YE. AFR_SAR species.

####### Published records.

Asir (Basilewsky 1979), Baha (El-Hawagry et al. 2013), Makkah (Britton 1948; Basilewsky 1979).

####### Remarks.

A frequent species, which was found only at low elevation (471 m) under stones and debris along the side of freshwater stream flowing through *Acacia* thorn woodlands. These adults were collected during autumn and winter by hand picking and by using a light trap. Mahmoud Abdel-Dayem identified this species.

#### Carabinae

##### Carabini

###### Calosoma imbricatum

Taxon classificationAnimaliaColeopteraCarabidae

Klug, 1832

####### Material examined.

892 m: 26.I.2015, LT, 1♀; 14.II.2014, LT, 2♂, 1♀; 15.II.2014, LT, 1♀; 12.XI.2015, LT, 1♂; 14.XI.2015, LT, 1♂. 1,225 m: 12.XI.2015, LT, 1♀. 1,325 m: 15.XI.2015, 1♀. 1,448 m: 03.XI.2013, LT, 1♀; 03.XI.2013, HP, 1♂. 1,474 m: 02.III.2015, LT, 1♀. 1,611 m: 15.II.2014, LT, 1♀; 21.IV.2014, LT, 1♀.

####### General distribution and zoogeography.

AE, BF, CV, DJ, DZ, EG, ER, ET, IQ, IR, KE, KW, LB, LY, ML, NE, OM, PK, QA, SA, SD, SN, SO, TD, YE. AFR_SAR species.

####### Published records.

Asir (Abdel-Dayem et al. 2018), Baha (El-Hawagry et al. 2013), Eastern Province (Heinertz 1979), Jizan and Makkah (Britton 1948), Riyadh (Heinertz 1979; Abdel-Dayem et al. 2017).

####### Remarks.

A rare species, which was collected during autumn, winter, and spring. The adults were collected by light trapping and hand picking in *Acacia* thorn woodlands and Barbary fig shrub community. Ali Elgharbawy identified this species.

###### Calosoma olivieri

Taxon classificationAnimaliaColeopteraCarabidae

Dejean, 1831

####### Material examined.

892 m: 15.II.2014, LT, ♀; 23.IV.2014, LT, 1♂; 13.XI.2015, LT, 1♂; 14.XI.2015, LT, 1♂, 1♀. 1,225 m: 15.II.2014, PT, 1♀. 1,325 m: 15.II.2014, LT, 1♂, 3♀; 15.II.2014, PT, 1♀; 15.XI.2015, LT, 2♀. 1,474 m: 15.II.2014, LT, 1♂. 1,611 m: 15.II.2014, LT, 3♂, 6♀.

####### General distribution and zoogeography.

AF, DZ, EG, IQ, IR, IT, JO, LY, MA, PK, SA, SY, TD, TM, TN, UZ, YE. PAL_SAR species.

####### Published records.

Makkah (Britton 1948, Mateu 1990); Riyadh (Heinertz 1979; Abdel–Dayem et al. 2017). New provincial records for Baha.

####### Remarks.

A frequent species that was collected during autumn, winter, and spring. The species was collected by light trapping and hand picking in *Acacia* thorn woodlands and Barbary fig shrub community. Ali Elgharbawy identified this species.

###### Calosoma senegalense

Taxon classificationAnimaliaColeopteraCarabidae

Dejean, 1831

####### Material examined

. 892 m: 15.XI.2015, LT, 1♀. 1,225 m: 14.XI.2015, LT, 1♂.

####### General distribution and zoogeography.

AO, BF, BI, BJ, BW, CD, CF, CG, CI, CM, CV, ER, ET, GA, GH, GM, GN, GQ, GW, KE, LR, LS, MG, ML, MR, MW, MZ, NA, NE, NG, RE, RW, SL, SN, SO, SZ, TD, TG, TZ, UG, ZA, ZM, ZW. New to Arabian Peninsula. This range exemplifies the AFR realm.

####### Remarks.

A rare species. The adults were collected only during autumn (November) by light trapping in an area dominated by *Acacia* trees. Mahmoud Abdel-Dayem identified this species.

#### Cicindelinae

##### Cicindelini

###### Calomera alboguttata

Taxon classificationAnimaliaColeopteraCarabidae

(Klug, 1832)

####### Material examined.

471 m: 03.III.2015, LT, 24 exs; 15.XI.2015, LT, 2 exs; 10.XII.2014, LT, 7 exs. 1,611 m: 02.III.2015, LT, 1 ex.

####### General distribution and zoogeography.

EG, ER, ET, KE, SA, SD, SO, YE. This range exemplifies the AFR realm.

####### Published records.

Makkah (Britton 1948). New record for Baha Province.

####### Remarks.

A frequent species collected during autumn and winter, with more individuals during late winter. These adult beetles were collected by light trapping in *Acacia* thorn woodlands and Barbary fig shrub community. Mahmoud Abdel-Dayem identified this species.

###### Cylindera rectangularis

Taxon classificationAnimaliaColeopteraCarabidae

(Klug, 1832)

####### Material examined.

1,474 m: 14.XI.2015, LT, 1 ex. 1,611 m: 23.VIII.2014, LT, 2 exs.

####### General distribution and zoogeography.

CD, ER, ET, KE, MW, MZ, SA, SD, SO, TZ, UG, YE. This range exemplifies the AFR realm.

####### Published records.

Asir (Abdel-Dayem et al. 2018), Makkah (Abdel-Dayem and Kippenhan 2013). New record for Baha Province.

####### Remarks.

A rare species. The adults were collected by light trapping during summer and autumn in *Acacia* thorn woodland community. Jürgen Wiesner identified this species.

###### Myriochila melancholica

Taxon classificationAnimaliaColeopteraCarabidae

(Fabricius 1798)

####### Material examined.

1,325 m: 21.IV.2014, LT, 1♂. 1,474 m: 23.VIII.2014, LT, 1♀.

####### General distribution and zoogeography.

AE, AF, AL, AM, AO, AZ, BF, BH, BJ, BW, CD, CF, CG, CI, CM, CN, CV, CY, CZ, DZ, EG, ER, ES, ET, FR, GA, GE, GH, GM, GN, GQ, GR, GW, IL, IN, IQ, IR, IT, JO, KE, KG, KZ, LB, LY, MA, MG, MW, MZ, NA, NG, NP, OM, PK, PT, QA, SA, SC, SD, SL, SN, SO, ST, SY, TD, TG, TJ, TM, TN, TR, TZ, UZ, YE, ZA, ZM, ZW. This range exemplifies AFR_MAD_ORR_PAL_SAR.

####### Published records.

Asir (Abdel-Dayem et al. 2018), Jizan and Najran (Britton 1948), Riyadh (Abdel-Dayem et al. 2017). New record for Baha Province.

####### Remarks.

A rare species. The two specimens were collected during summer and spring by light trapping in *Acacia* thorn woodlands. Mahmoud Abdel-Dayem identified this species.

###### Myriochila nudopectoralis

Taxon classificationAnimaliaColeopteraCarabidae

(W. Horn, 1903)

####### Material examined.

1,325 m: 02.IX.2015, LT, 1♂, 1♀.

####### General distribution and zoogeography.

ET, OM, YE. New country record. This range exemplifies the AFR realm.

####### Remarks.

A rare species that was collected during summer by light trapping from *Acacia* thorn woodlands. Jürgen Wiesner identified this species.

#### Harpalinae

##### Chlaeniini

###### Chlaenius canariensis seminitidus

Taxon classificationAnimaliaColeopteraCarabidae

Chaudoir, 1856

####### Material examined.

892 m: 15.II.2014, LT, 2♂; 21.IV.2014, LT, 1♂, 1♀; 14.XI.2015, LT, 1♂. 1,225 m: 15.II.2014, LT, 1♀; 14.XI.2015, LT, 1♂. 1,325 m: 15.II.2015, LT, 2♂; 3.VI.2014, LT, 1♂. 1,611 m: 15.II.2014, LT, 2♀; 21.IV.2014, LT, 3♂, 1♀. 1,666 m: 27.I.2014, LT, 1♂.

####### General distribution and zoogeography.

AE, DZ, EG, IL, IQ, IR, JO, LB, LY, MA, SA, SY, TN, TR. This range exemplifies the SAR realm.

####### Published records.

Asir (Abdel-Dayem et al. 2018). New record for Baha Province.

####### Remarks.

A frequent species that was found at all elevation levels in the SANR in *Acacia* thorn woodlands and Barbary fig communities. The adults were collected by light trapping during autumn, winter, and spring. Erich Kirschenhofer identified this subspecies.

###### Chlaenius flavipes

Taxon classificationAnimaliaColeopteraCarabidae

Ménétriés, 1832

####### Material examined.

1,008 m: 08.XII.2014, HP, 1♂.

####### General distribution and zoogeography.

AF, AL, AM, AZ, BA, BG, GE, GR, HR, HU, IN, IQ, IR, KG, KZ, MD, MK, NP, PK, RO, RU, SA (Abdel-Dayem et al. 2017), TJ, TM, TR, UA, UZ. ORR_PAL_SAR species.

####### Published records.

Asir and Riyadh (Abdel-Dayem et al. 2017, 2018). New record for Baha Province.

####### Remarks.

A rare species that was collected during autumn. The only adult specimen was collected by hand under a stone at the edges of freshwater pools in *Acacia* thorn woodlands. Erich Kirschenhofer identified this species.

###### Chlaenius laeviplaga saudiarabica

Taxon classificationAnimaliaColeopteraCarabidae

Mandl, 1980

####### Material examined.

471 m: 10.XII.2014, HP, 9♂, 5♀. 892 m: 10.XII.2014, LT, 1♂, 1♀.

####### General distribution and zoogeography.

AE, SA. END_AR species.

####### Published records.

Asir (Mandl 1980; Abdel-Dayem et al. 2018). New record for Baha Province.

####### Remarks.

A frequent species that was collected during autumn. The adults were found under stones along the margins of a freshwater stream and collected by hand. Additional adults collected at night by using lights. Erich Kirschenhofer identified this subspecies.

###### Chlaenius pachys

Taxon classificationAnimaliaColeopteraCarabidae

Chaudoir, 1876

####### Material examined.

471 m: 10.XII. 2014, HP, 2♀.

####### General distribution and zoogeography.

ER, ET, JO (Nasir and Katbeh-Bader 2017), SD, SO, SA, YE. AFR species.

####### Published records.

Jizan and Makkah (Britton 1948). New provincial records for Baha.

####### Remarks.

A rare species that was collected during late autumn. The two adults were found under stones along the edge of freshwater stream collected by hand picking. Erich Kirschenhofer identified this species.

##### Cyclosomini

###### Tetragonoderus arcuatus

Taxon classificationAnimaliaColeopteraCarabidae

Dejean, 1829

####### Material examined.

1611 m, 21.IV.2014, LT, 1♂; 03.VI.2014, LT, 1♀.

####### General distribution and zoogeography.

BD, CN, EG, ET, IL (Assmann et al. 2015), IN, IQ, LA, MM, NE, NP, OM, PK, SD, TD, YE. AFR_ORR_SAR species.

####### Published records.

known only from Baha Province (El-Hawagry et al. 2013).

####### Remarks.

A rare species that was collected during spring by light trapping in a Barbary fig shrub community. Mahmoud Abdel-Dayem identified this species.

###### Tetragonoderus quadrum

Taxon classificationAnimaliaColeopteraCarabidae

(Fabricius, 1792)

####### Material examined.

892 m: 14.II.2014, HP, 1♂; 15.II.2014, LT, 1♀. 1,325 m: 02.III.2015, LT, 1♀; 03.VI.2014, LT, 1♂; 05.VI.2014, PT, 1♀. 1563 m, 17.X.2014, LT,1♀.

####### General distribution and zoogeography.

ER, ET, GM, MR, SA, SN, SO, TD. AFR species.

####### Published records.

Jizan (Mateu 1990). New record for Baha Province.

####### Remarks.

A rare species which was collected during autumn, winter, and spring from *Acacia* thorn woodlands and Barbary fig shrub communities. Mahmoud Abdel-Dayem identified this species.

##### Harpalini

###### Amblystomus orpheus

Taxon classificationAnimaliaColeopteraCarabidae

(LaFerté-Sénectěre, 1853)

####### Material examined.

825 m: 14.XI.2015, LT, 2 exs; 15.XI.2015, LT, 1 ex. 892 m: 17.X.2014, LT, 1 ex; 14.XI.2015, LT, 1ex; 1,225 m: 2.IX.2015, LT, 2 exs. 1,325 m: 2.IX.2015, LT, 4 exs; 17.X.2014, LT, 1ex; 15.XI.2015, LT, 3 exs. 1,474 m: 14.XI.2015, LT, 1ex. 1,563 m: 20.IV.2014, LT, 2 exs; 14.XI.2015, LT, 4 exs. 1,611 m: 2.IX.2015, LT, 1 ex.

####### General distribution and zoogeography.

AE, AO, BF, BI, CD, CF, CM, CV, ER, ET, KE, ML, MR, MW, MZ, NA, NE, SA (Abdel-Dayem et al. 2018), SN, SO, TD, TZ, UG, YE, ZA, ZW. AFR species.

####### Published records.

Asir (Abdel-Dayem et al. 2018). New provincial records for Baha.

####### Remarks.

A frequent species that was collected during late summer (September) to autumn, and spring. The adult specimens were collected only using light traps in *Acacia* thorn woodlands and cactus communities. David Wrase identified this species.

###### Amblystomus

Taxon classificationAnimaliaColeopteraCarabidae

sp.

####### Material examined.

892 m: 16.X.214, LT, 1♂, 1♀; 15.XI.2015, LT, 1♂, 1♀; 08.XII.2014, LT, 1♀.1,611 m: 21.IX.2015, LT, 1♀.

####### Remarks.

A rare species that was collected during late summer and autumn. The above specimens were collected by light traps set at lower altitude in *Acacia* thorn woodlands. David Wrase and Boris Kataev identified this taxon.

###### Anthracus angusticollis

Taxon classificationAnimaliaColeopteraCarabidae

(Péringuey, 1908)

####### Material examined.

1,474 m: 17.X.2014, LT, 1♂.

####### General distribution and zoogeography.

AE, CD, ET, GM, MG, MR, NA, SN, SO, TD, TZ, ZA. New country record. AFR species.

**Remarks.** A rare species. The single above male was collected during autumn at light in *Acacia* thorn woodlands. Bernd Jaeger identified this species.

###### Crasodactylus punctatus

Taxon classificationAnimaliaColeopteraCarabidae

Guerin-Meneville, 1847

####### Material examined.

851 m: 15.II.2014, LT, 2 exs. 892 m: 26.I.2015, LT, 1 ex; 15.II.2014, LT, 2 exs; 20.IV.2014, LT, 1 ex; 15.X.2014, LT, 4 exs; 17.X.2014, LT, 3 exs; 18.X.2014, LT, 2 exs; 14.XI.2015, LT, 3 exs; 15.XI.2015, LT, 1 ex; 07.XII.2014, LT, 1 ex. 1,225 m: 02.IX.2015, LT, 1 ex; 17.X.2014, LT, 3 exs; 14.XI.2015, LT, 1 ex. 1,325 m: 17.X.2014, LT, 14 exs; 18.X.2014, PT, 1 ex; 15.XI.2015, LT, 9 exs. 1,474 m: 17.X.2014, LT, 1 ex; 18.X.2014, PT, 3 exs. 1,448 m: 03.XI.2013, LT, 31 exs. 1,563 m: 14.XI.2015, LT, 1 ex. 1,611 m: 02.IX.2015, LT, 1 ex. 1,666 m: 17.X.2014, LT, 10 exs.

####### General distribution and zoogeography.

AF, CD, DJ, ER, ET, KE, IQ, IR, NE, OM, PK, SA (Abdel-Dayem et al. 2018), SO, TD, TN, YE (including Socotra). AFR_SAR species.

####### Published records.

Asir (Abdel-Dayem et al. 2018). New provincial records for Baha.

####### Remarks.

A common species that was collected between an altitude of 851–1666 m in *Acacia* thorn woodlands and a Barbary fig shrub community using both light traps and pitfall traps. The highest number of individuals were collected during autumn. David Wrase identified this species.

###### Harpalus impressus

Taxon classificationAnimaliaColeopteraCarabidae

Roth, 1851

####### Material examined.

892 m: 23.IV.2014, LT, 1 ex. 1,474 m: 2.XI.2013, HP, 3 exs.

####### General distribution and zoogeography.

ER, ET, SD, SA, YE, UG. AFR species.

####### Published records.

Asir (Basilewsky 1979; Abdel-Dayem et al. 2018).

####### Remarks.

A rare species that was collected during summer and autumn from *Acacia* thorn woodlands. David Wrase identified this species.

###### Harpalus tenebrosus tenebrosus

Taxon classificationAnimaliaColeopteraCarabidae

Dejean, 1829

####### Material examined.

1,225 m: 02.IX.2015, LT, 1♂, 2♀. 1,666 m: 02.IX.2015, LT, 1♀.

####### General distribution and zoogeography.

AF, AL, AM, AT, AZ, BA, BE, BG, CH, CY, CZ, DE, DZ, EG, ES, FR, GB, GE, GR, HR, HU, IL, IQ, IR, IT, JO, MA, MD, ME, MK, MR, OM, PK, PL, PT, RO, RS, RU, SA (Abdel-Dayem et al. 2018), SI, SK, SY, TJ, TM, TN, TR, UA, UZ. PAL_SAR subspecies.

####### Published records.

Asia (Abdel-Dayem et al. 2018). New provincial records for Baha.

####### Remarks.

A rare species collected during late summer by light trapping in *Acacia* thorn woodlands and Barbary fig communities. David Wrase identified this species.

###### Progonochaetus planicollis

Taxon classificationAnimaliaColeopteraCarabidae

(Putzeys, 1880)

####### Material examined.

471 m: 10.XII. 2014, HP, 1♀. 892 m: 26.I.2015, LT, 1♂, 1♀; 14.II.2014, LT, 3♂, 7♀; 15.II.2014, LT, 4♂, 5♀; 16.II.2014, LT, 1♂; 20.IV.2014, LT, 1♂; 21.IV.2014, LT, 1♀; 23.IV.2014, LT, 1♀; 15.XI.2015, LT, 1♀; 07.XII.2014, LT, 1♀; 08.XII.2014, LT, 1♂, 3♀; 10.XII.2014, LT, 1♂. 1,225 m: 27.I.2015, LT, 1♀. 1,325 m: 15.II.2014, LT, 4♂; 02.III.2015, LT, 1♀. 1,474 m: 15.II.2014, LT, 1♂. 1,448 m: 03.XI.2013, LT, 1♂. 1,563 m: 15.II.2014, LT, 1♀. 1,611 m: 15.II.2014, LT, 1♀; 21.IV.2014, LT, 1ex. 1,666 m: 21.IV.2014, LT, 1ex.

####### General distribution and zoogeography.

AO, BF, CM, CD, CF, CG, CI, DJ, ER, ET, GA, GH, GN, GQ, GW, KE, ML, MZ, NA, NG, RW, SA, SN, SO, TD, TZ, YE, ZM, ZW. AFR species.

####### Published records.

Asir (Basilewsky 1979; Abdel-Dayem et al. 2018). New record for Baha Province.

####### Remarks.

A frequent species that was collected during all seasons with more individuals collected during winter. The adults were collected by light trapping and hand picking in *Acacia* thorn woodlands and Barbary fig communities. David Wrase identified this species.

###### Siopelus quadraticollis

Taxon classificationAnimaliaColeopteraCarabidae

(Putzeys in Chaudoir, 1878)

####### Material examined.

892 m: 26.I.2015, LT, 1ex; 15.II.2014, LT, 1♂, 2♀; 18.X.2014, LT, 1♂, 2♀; 14.XI.2015, LT, 2♂, 5♀; 07.XII.2014, LT, 1♂, 1♀. 1,225 m: 15. II.2014, HP, 1♂; 17.X.2014, LT, 6♂, 4♀; 12.XI.2015, LT, 1♂. 1,325 m: 17.X.2014, LT, 14♀; 18.X.204, PT, 1♀; 15.XI.2015, LT, 2♀; 08.XII.2014, HP, 2♀. 1388 m: 08.XII.2014, HP, 1♀. 1,448 m: 03.XI.2013, HP, 3♀. 1,474 m: 17.X.2014, LT, 1♂; 18.X.2014, PT, 3♀; 02.IX.2015, LT, 2♀. 1,563 m: 02.IX.2015, LT, 3♀; 17.X.2014, LT, 1♂, 2♀. 1,611 m: 02.IX.2015, LT, 1♀.

####### General distribution and zoogeography.

ET, SA (Abdel-Dayem et al. 2018), TZ. AFR species.

####### Published records.

Asir (Abdel-Dayem et al. 2018). New record for Baha Province.

####### Remarks.

A common species that was collected during late summer, autumn, and winter. The adults were collected by light and pitfall trapping in *Acacia* thorn woodlands and Barbary fig communities. David Wrase identified this species.

##### Lebiini

###### Apristus arabicus

Taxon classificationAnimaliaColeopteraCarabidae

Mateu, 1986

####### Material examined.

1,325 m: 17.X.2014, LT, 1 ex. 1,474 m: 03.XI.2013, HP, 1 ex.

####### General distribution and zoogeography.

AE, IQ, SA. SAR species.

####### Published records.

Asir (Abdel-Dayem et al. 2018), Makkah (Mateu 1986). New record for Baha Province.

####### Remarks.

A rare species collected during autumn from *Acacia* thorn woodlands by light trapping and hand picking. Ron Felix identified this species.

###### Calodromius mayeti

Taxon classificationAnimaliaColeopteraCarabidae

(Bedel, 1907)

####### Material examined.

825 m: 13.XI.2015, LT, 1♂, 1♀; 15.XI.2015, LT, 1♂, 2♀. 851 m: 15.XI.2015, LT, 1♀. 892 m: 26.I.2015, LT, 1♀; 16.II.2014, LT, 2♂; 13.XI.2015, LT, 1♀. 1,225 m: 20.IV.2014, LT, 2♀; 12.XI.2015, LT, 1♂, 1♀. 1,325 m: 02.III.2015, LT, 1♂, 2♀; 14.XI.2015, LT, 1♂; 17.X.2014, LT, 1♀. 1,474 m: 17.X.2014, LT, 1♀.

####### General distribution and zoogeography.

AE, DZ, IR, JO (Nasir and Katbeh-Bader 2017), LY, MA, SA, TN. SAR species.

####### Published records.

Asir and Riyadh (Abdel-Dayem et al. 2017, 2018), Baha (Rasool et al. 2018b), Madinah and Makkah (Mateu 1986).

####### Remarks.

A frequent species that was found in *Acacia* thorn woodlands during the four seasons of year, but peak populations occurred during autumn. The adults were collected by light trapping and hand picking. Ron Felix identified this species.

###### Dromius buettikeri

Taxon classificationAnimaliaColeopteraCarabidae

Mateu, 1990

####### Material examined.

825 m: 13.XI.2015, LT, 1♀; 15.XI.2015, LT, 1♂. 851 m: 15.XI.2015, LT, 1♀. 892 m: 12.XI.2015, LT, 1♂, 1♀. 1,325 m: 27.I.2015, LT, 1♀; 14.XI.2015, LT, 1♂, 2♀; 15.XI.2015, LT, 1♀. 1,474 m: 27.I.2014, LT, 1♀; 15.II.2014, LT, 1♂, 1♀; 14.XI.2015, LT, 1♂; 08.XII.2014, LT, 1♂. 1,563 m: 27.I.2015, LT, 1♂. 1,611 m: 27.I.2015, LT, 1♀. 1,666 m: 27.I.2015, LT, 1♀.

####### General distribution and zoogeography.

SA. END_SA species.

####### Published records.

Asir and Baha (Rasool et al. 2018b), Riyadh (Mateu 1990).

####### Remarks.

A frequent species. The adults were collected during autumn and winter from *Acacia* thorn woodlands and Barbary fig communities. The specimens were attracted only to light trap. Ron Felix identified this species.

###### Eremolestes sulcatus

Taxon classificationAnimaliaColeopteraCarabidae

(Chaudoir, 1876)

####### Material examined.

1,474 m: 23.IV.2014, PT, 1♂. 1562 m: 03.XI.2013, HP, 1♂, 1♀.

####### General distribution and zoogeography.

DJ, DZ, ER, ET, SA, SD, TD. This range exemplifies AFR realm.

####### Published records.

Najran and Riyadh (Mateu 1979, 1986). New record for Baha Province.

####### Remarks.

A rare species that was found during autumn and spring collected by pitfall trapping and hand picking in *Acacia* thorn woodlands and Barbary fig shrub communities. Ron Felix identified this species.

###### Lebia auberti

Taxon classificationAnimaliaColeopteraCarabidae

Fairmaire, 1892

####### Material examined.

1,225 m: 08.XII.20114, LT, 1♀. 1,325 m: 02.IX.2015, LT, 1♂.

####### General distribution and zoogeography.

DJ, SA (Rasool et al. 2018a). This range exemplifies AFR realm.

####### Published records.

Asir (Abdel-Dayem et al. 2018, Rasool et al. 2018), Baha (Rasool et al. 2018a), Riyadh (Mateu 1986).

####### Remarks.

A rare species with each sex represented by a single specimen collected from *Acacia* thorn woodlands during late summer and early autumn. Alexander Anichtchenko and Ron Felix identified this species.

###### Lebia nilotica

Taxon classificationAnimaliaColeopteraCarabidae

Chaudoir, 1871

####### Material examined.

825 m: 14.XI.2015, LT, 7♂, 10♀; 15.XI.2015, LT, 4♂, 7♀. 892 m: 26.I.2015, LT, 2♂, 3♀; 14.II.2014, LT, 2♂, 3♀; 15.II.2014, LT, 2♂, 2♀; 20.IV.2014, LT, 4♂, 3♀; 21.IV.2014, LT, 1♂, 1♀; 16.X.2014, LT, 1♂; 18.X.2014, LT, 2♂, 4♀; 14.XI.2015, HP, 20♂, 26♀; 14.XI.2015, LT, 1♀; 15.XI.2015, LT, 2♂, 4♀; 09.XII.2014, LT, 1♀; 10.XII.2014, LT, 1♀; 11.XII.2014, LT, 1♀. 1,225 m: 27.I.2015, LT, 1♀; 15.II.2014, HP, 3♀; 15.II.2014, LT, 3♀; 21.IV.2014, LT, 3♂, 6♀; 02.IX.2015, LT, 19♂, 31♀; 14.XI.2015, LT, 3♀; 15.XI.2015, LT, 1♀. 1,325 m: 27.I.2015, LT, 1♀; 15.II.2014, LT, 1♂, 5♀; 15.II.2014, MT, 1♀; 21.IV.2014, LT, 4♂, 9♀; 03.VI.2014, LT, 1♂, 1♀; 23.VIII.2014, LT, 2♀; 02.IX.2015, LT, 14♂, 25♀; 15.XI.2015, LT, 4♂, 6♀. 1,474 m: 15.II.2014, LT, 1♂, 3♀; 21.IV.2014, LT, 2♂, 3♀; 05.V.2015, LT, 1♀; 23.VIII.2014, LT, 1♀; 02.IX.2015, LT, 41exs; 14.XI.2015, LT, 1♀. 1,563 m: 05.V.2015, MT, 2exs; 02.IX.2015, LT, 96exs; 17.X.2014, LT, 1♀. 1,611 m: 21.IV.2014, LT, 4♂, 4♀; 03.VI.2014, LT, 1♂; 03.VI.2014, SW, 1♂; 02.IX.2015, LT, 62exs; 17.X.2014, LT, 1♀; 15.XI.2015, LT, 1♀. 1,666 m: 21.IV.2014, LT, 2♂; 03.VI.2014, LT, 1♂, 2♀; 02.IX.2015, LT, 217exs.

####### General distribution and zoogeography.

EG, IQ, SA. This range exemplifies SAR realm.

####### Published records.

Asir (Mateu 1979, Abdel-Dayem et al. 2018, Rasool et al. 2018), Baha and Jizan (Rasool et al. 2018a).

####### Remarks.

An abundant species that was recorded during all seasons of the year from a wide altitudinal range (471–1666 m). Most individuals were collected during late summer (September). Mahmoud Abdel-Dayem and Iftekhar Rasool identified this species.

###### Lebia raeesae

Taxon classificationAnimaliaColeopteraCarabidae

Rasool, Abdel-Dayem & Felix, 2018

####### Material examined.

1,611 m: 21.IV.2014, LT, 1♀. 1,666 m: 27.I.2015, LT, 1♀; 02.III.2015, LT, 1♀; 21.IV.2014, LT, 1♀; 23.VIII.2014, LT, 1♂; 02.IX.2015, LT, 3♂, 4♀.

####### General distribution and zoogeography.

SA, YE (Rasool et al. 2018a). This range exemplifies SAR realm.

####### Published records.

Asir and Baha (Rasool et al. 2018a).

####### Remarks.

A rare species that was collected during spring, summer, and winter by light trapping in Barbary fig shrubs communities. Iftekhar Rasool, Mahmoud Abdel-Dayem and Ron Felix identified this species.

###### Matabele arabica

Taxon classificationAnimaliaColeopteraCarabidae

Mateu, 1986

####### Material examined.

1,225 m: 14.XI.2015, LT, 1♀. 1,563 m: 23.VII.2015, LT, 1♀.

####### General distribution and zoogeography.

OM, SA. This range exemplifies SAR realm.

####### Published records.

Asir (Mateu 1986), Baha (Rasool et al. 2018a).

####### Remarks.

A rare species that was collected during autumn and summer, represented by a single specimen during each season. Ron Felix identified this species.

###### Merizomena buettikeri

Taxon classificationAnimaliaColeopteraCarabidae

(Mateu, 1986)

####### Material examined.

825 m: 13.XI.2015, LT, 2♀; 15.XI.2015, LT, 20 exs. 851 m: 14.XI.2015, LT, 23 exs; 15.XI.2015, LT, 1♂, 2♀. 892 m: 13.XI.2015, LT, 1♀; 15.XI.2015, LT, 24 exs. 1,225 m: 17.X.2015, LT, 1♀; 12.XI.2015, LT, 1♂, 1♀; 14.XI.2015, LT, 1♂, 2♀;. 1611m: 23.VIII.2014, LT, 1♀.

####### General distribution and zoogeography.

SA. END_SA species.

####### Published records.

Asir and Riyadh (Abdel-Dayem et al. 2017, 2018), Madinah and Najran (Mateu 1986). New record for Baha Province.

####### Remarks.

A common species with more individuals collected during autumn from *Acacia* thorn woodlands and relatively few individuals during late summer from a Barbary fig shrub community. Iftekhar Rasool, Mahmoud Abdel-Dayem and Ron Felix identified this species.

###### Metadromius arabicus

Taxon classificationAnimaliaColeopteraCarabidae

Mateu, 1979

####### Material examined.

825 m: 13.XI.2015, LT, 2♂, 4♀; 15.XI.2015, LT, 5♂, 3♀. 851 m: 14.XI.2015, LT, 1♂; 15.XI.2015, LT, 2♂, 2♀. 892 m: 16.X.2015, LT, 1♂; 17.X.2014, LT, 6♀; 15.XI.2015, LT, 1♂, 1♀. 1,225 m: 02.III.2015, LT, 1♂, 1♀; 02.IX.2015, LT, 2♀; 17.X.2014, LT, 1♂, 2♀; 15.XI.2015, LT, 1♀. 1,325 m: 27.I.2015, LT, 1♀; 02.III.2015, LT, 1♂; 02.IX.2015, LT, 2♂, 1♀; 17.X.2014, LT, 1♂, 1♀; 14.XI.2015, LT, 3♂, 5♀. 1,474 m: 15.II.2014, LT, 1♂, 2♀; 02.IX.2015, LT, 38♂, 52♀; 17.X.2014, LT, 3♂, 2♀. 1562 m: 03.XI.2013, HP, 1♀. 1,563 m: 21.IV.2014, LT, 2♂, 4♀; 02.IX.2015, LT., 51♂, 71♀; 17.X.2014, LT, 3♂, 6♀; 14.XI.2015, LT, 2♂; 18.XI.2015, LT, 2♀. 1,611 m: 27.I.2015, LT, 1♂; 02.IX.2015, LT, 122♂, 146♀; 17.X.2014, LT, 4♂, 2♀. 1,666 m: 02.III.2015, LT, 3♂, 5♀; 05.V.2015, LT, 2♀; 02.IX.2015, LT, 21♂, 29♀.

####### General distribution and zoogeography.

AE, IR, SA. This range exemplifies SAR realm.

####### Published records.

Asir and Baha (Abdel-Dayem et al. 2018; Rasool et al. 2018b), Riyadh (Mateu 1979).

####### Remarks.

An abundant species that was collected during all four seasons with most specimens caught during late summer (September). The adults of this species were only collected using light traps. Iftekhar Rasool, Mahmoud Abdel-Dayem and Ron Felix identified this species.

###### Metadromius brittoni

Taxon classificationAnimaliaColeopteraCarabidae

(Basilewsky, 1948)

####### Material examined.

471 m: 3.III.2015, LT, 1♀; 10.XII.2014, LT, 1♀. 825 m: 13.XI.2015, LT, 5 exs; 15.XI.2015, LT, 7 exs. 851 m: 13.XI.2015, LT, 1♂; 15.XI.2015, LT, 17 exs. 892 m: 16.II.2014, LT, 1♀; 03.III.2015, LT, 2♀; 23.IV.2014, LT, 12♂, 8♀; 23.VIII.2014, LT, 1♂; 17.X.2014, LT, 24♂, 15♀. 16.X.2014, LT, 2♂, 4♀. 1,225 m: 27.I.2015, LT, 1♂; 02.III.2015, LT, 5♂, 3♀; 21.IV.2014, LT, 17♂, 10♀; 05.V.2015, LT, 9 exs; 03.VI.2014, LT, 21♂, 17♀; 23.VIII.2014, LT, 8♂, 5♀; 24.VIII.2014, LT, 1♂; 02.IX.2015, PT, 1 ex; 17.X.2014, LT, 2♂, 3♀; 18.X.2014, PT, 1♀. 1,325 m: 02.III.2015, LT, 6 exs; 21.IV.2014, LT, 2♂, 3♀; 05.V.2015, LT, 1 ex; 23.VIII.2014, LT, 13♂, 10♀; 02.IX.2015, LT, 3 exs; 17.X.2014, LT, 2♂, 5♀. 1,474 m: 20.IV.2014, LT, 1 ex; 23.VIII.2014, LT, 1♂; 02.IX.2015, LT, 1♂; 17.X.2014, LT, 1♂. 1562 m, 03.XI.2013, HP, 1♂. 1563 m, 21.IV.2014, LT, 1♂, 1♀; 03.VI.2014, LT, 3 exs. 1611 m 23.VIII.2014, VC, 1♂; 17.X.2014, LT, 1♂; 02.IX.2015, LT, 2 exs; 18.X.2014, PT, 1♀.

####### General distribution and zoogeography.

JO, SA, YE. This range exemplifies SAR realm.

####### Published records.

Asir, Baha and Jizan (Abdel-Dayem et al. 2018; Rasool et al. 2018b), Riyadh (Mateu 1979).

####### Remarks.

A common species that was collected during all four seasons from different altitudinal ranges (471–1611 m) of the SANR. Iftekhar Rasool, Mahmoud Abdel-Dayem and Ron Felix identified this species.

###### Metadromius

Taxon classificationAnimaliaColeopteraCarabidae

sp.

####### Material examined.

471 m: 03.III.2015, LT, 1♂. 1,325 m: 02.III.2015, LT, 1♀.

####### Remarks.

A rare species that was collected during late winter. It is similar to *M.ephippiatus* (Fairmaire, 1884), which is known from North Africa (DZ, MA, TN) (Löbl and Löbl 2017). However, these specimens along with SA specimens identified by Mateu (1986) are rather different from *M.ephippiatus*. The specimens from SA probably represent a new species. These specimens have been included in a current taxonomic revision of the Middle East *Metadromius* (R. Felix, personal communication). Ron Felix identified this species.

###### Microlestes discoidalis

Taxon classificationAnimaliaColeopteraCarabidae

(Fairmaire, 1892)

####### Material examined.

825 m: 13.XI.2015, LT, 1♂; 15.XI.2015, LT, 1♂. 851 m: 15.XI.2015, LT, 2♂, 1♀; 892 m: 26.I.2015, LT, 2♂, 1♀; 15.II.2014, LT, 3♂, 8♀; 23.IV.2014, LT, 1♂; 1,563 m: 21.IV.2014, LT, 2♂. 1,611 m: 02.IX.2015, LT, 1♂, 2♀.

####### General distribution and zoogeography.

AE, AF, BD, CD, CV, DJ, ER, ET, IL, IN, IR, JO (Nasir and Katbeh-Bader 2017), KE, MR, NE, OM, SA, SD, SO, TD, TR, TZ, YE. AFR_ORR_SAR species.

####### Published records.

Asir, Baha, Jizan and Riyadh (Abdel-Dayem et al. 2017; 2018; Rasool et al. 2018b), Makkah (Britton 1948; Mateu 1986).

####### Remarks.

A frequent species that was collected during all seasons of the year with more individuals during the winter collecting dates. Iftekhar Rasool, Mahmoud Abdel-Dayem and Ron Felix identified this species.

###### Microlestes infuscatus fragilis

Taxon classificationAnimaliaColeopteraCarabidae

Mateu, 1956

####### Material examined.

892 m, 23.IV.2014, LT, 7♂, 9♀. 1,325 m: 23.VIII.2014, LT, 1♀. 1,474 m: 02.IX.2015, LT, 1♀. 1,666 m: 02.II.2015, PT, 1♀; 23.VIII.2014, LT, 1♀; 02.IX.2015, LT, 1♂; 15.XI.2015, PT, 1♀.

####### General distribution and zoogeography.

AF, SA, YE. This range exemplifies SAR realm.

####### Published records.

Asir, Baha and Jizan (Mateu 1979; Abdel-Dayem et al. 2018; Rasool et al. 2018b).

####### Remarks.

A frequent species, which recorded during all seasons with highest numbers collected during spring (April). Iftekhar Rasool, Mahmoud Abdel-Dayem and Ron Felix identified this species.

###### Pseudomesolestes quadriguttatus

Taxon classificationAnimaliaColeopteraCarabidae

Mateu, 1979

####### Material examined.

851 m: 15.XI.2015, LT, 1♀. 892 m: 26.I.2015, LT, 1♀; 17.X.2014, LT, 1♂; 18.X.2014, LT, 1♂, 2♀; 15.XI.2015, LT, 1♀. 1,325 m: 02.III.2015, LT, 1♂. 1,563 m: 17.X.2014, LT, 1♀.

####### General distribution and zoogeography.

SA. END_SA species.

####### Published records.

Asir and Baha (Rasool et al. 2018b), Riyadh (Mateu 1979).

####### Remarks.

A rare species that was collected by light trapping during late summer (September), autumn, and winter. Iftekhar Rasool, Mahmoud Abdel-Dayem and Ron Felix identified this species.

###### Singilis discoidalis

Taxon classificationAnimaliaColeopteraCarabidae

(Mateu, 1986)

####### Material examined.

851 m: 15.XI.2015, LT, 1♂. 1,225 m: 21.IV.2014, LT, 1♂; 03.VI.2014,1♀; 02.III.2015, 1♀. 1,325 m: 03.VI.2014, LT, 1♂, 2♀; 27.VII.2015, LT, 1♀.

####### General distribution and zoogeography.

EG, IL, SA, YE. This range exemplifies SAR realm.

####### Published records.

Asir (Abdel-Dayem et al. 2018), Madinah, Makkah and Najran (Mateu 1986). New record for Baha Province.

####### Remarks.

A rare species that was caught by light trapping during the different seasons from *Acacia* thorn woodlands. Alexander Anichtchenko identified this species.

###### Singilis

Taxon classificationAnimaliaColeopteraCarabidae

sp.

####### Material examined.

851 m: 15.XI.2015, LT, 1♀.

####### Remarks.

A rare species that was collected by light trapping in *Acacia* thorn woodlands during autumn. This unidentified species is closely related to *S.cordiger* (Peringuey, 1896), which is known from NA, ZA and ZW. Unfortunately, only a single female was collected, and males are needed for identification. Alexander Anichtchenko identified this species.

###### Syntomus submaculatus

Taxon classificationAnimaliaColeopteraCarabidae

(Wollaston, 1861)

####### Material examined.

892 m: 21.IV.2014, LT, 1♀. 1,225 m: 28.VIII.2014, LT, 1♀; 08.XII.2014, VC, 2♀. 1,325 m: 15.XI.2015, PT, 1♀. 1388 m: 08.XII.2014, HP, 5♂ 6♀. 1,474 m: 08.XII.2014, VC, 1♀. 1562 m: 03.XI.2013, 2♂ 4♀. 1,563 m: 05.VI.2014, PT, 1♀.

####### General distribution and zoogeography.

CV, MR, SA TD, YE. This range exemplifies SAR realm.

####### Published records.

Jizan (Mateu 1986). New record for Baha Province.

####### Remarks.

A frequent species that was collected during spring, summer, and autumn. Iftekhar Rasool, Mahmoud Abdel-Dayem and Ron Felix identified this species.

###### Zolotarevskyella rhytidera

Taxon classificationAnimaliaColeopteraCarabidae

(Chaudoir, 1876)

####### Material examined.

1,474 m: 18.X.2014, PT, 1♀. 1562 m: 03.XI.2013, HP, 1♀.

####### General distribution and zoogeography.

CD, CV, ER, ET, GM, ML, NE, SA, SD, SN, TD, YE. This range exemplifies AFR realm.

####### Published records.

Asir, Baha and Jizan (Rasool et al. 2018b), Najran (Mateu 1986).

####### Remarks.

A rare species collected during the autumn from *Acacia* thorn woodlands and a Barbary fig shrub community. Iftekhar Rasool, Mahmoud Abdel-Dayem and Ron Felix identified this species.

##### Perigonini

###### Perigona nigriceps

Taxon classificationAnimaliaColeopteraCarabidae

(Dejean, 1831)

####### Material examined.

1,225 m: 02.III.2015, LT, 2 exs; 24.VIII.2014, LT, 1 ex; 02.IX.2015, LT, 1 ex; 17.X.2014, LT, 1 ex. 1,325 m: 02.III.2015, LT, 5 exs; 23.VIII.2014, LT, 1 ex; 14.XI.2015, LT, 1 ex; 15.XI.2015, LT, 2 exs. 1,563 m: 05.V.2015, LT, 1 ex. 1,611 m: 02.III.2015, LT, 1 ex; 05.V.2015, LT, 2 exs; 02.IX.2015, LT, 130 exs. 1,666 m: 02.IX.2015, LT, 99 exs; 02.IX.2015, PT, 1 ex.

####### General distribution and zoogeography.

AE, AO, AT, AU, AZ, BA, BB, BE, BG, BI, CA, CD, CG, CH, CI, CM, CN, CR, CU, CZ, DE, DK, ES, ET, FI, FR, GB, GH, GN, GP, GR, HR, HU, ID, IN, IQ, IR, IT, JP, KE, KH, KM, KP, KR, LK, LR, LU, LV, MD, MG, MM, MQ, MU, NC, NG, NL, NO, NZ, PG, PH, PL, PR, PT, RE, RU, RW, SA (Abdel-Dayem et al. 2018), SB, SC, SE, SI, SK, SL, SN, ST, TH, TW, TZ, UG, US, VN, YE, ZA, ZM, ZW. COS species.

####### Published records.

Only reported from Asir (Abdel-Dayem et al. 2018). New record for Baha Province.

####### Remarks.

A common species that was collected during all seasons with the highest number of individuals caught during late summer (September). The adults were collected mainly by light trapping in *Acacia* thorn woodlands and Barbary fig shrub communities. Mahmoud Abdel-Dayem identified this species.

#### Melaeninae

##### Cymbionotini

###### Cymbionotum microphthalmum

Taxon classificationAnimaliaColeopteraCarabidae

Chaudoir, 1876

####### Material examined.

892 m: 23.IV.2014, LT, 1 ex.

####### General distribution and zoogeography.

ET, NE, SA, SN, TR, YE. AFR_SAR species.

####### Published records.

Known only from Eastern Province (Basilewsky 1979). New provincial records for Baha.

####### Remarks.

A rare species that was collected during spring from *Acacia* thorn woodlands community. Mahmoud Abdel-Dayem identified this species.

#### Panagaeinae

##### Panagaeini

###### Microcosmodes arabicus

Taxon classificationAnimaliaColeopteraCarabidae

Häckel & Azadbakhsh, 2016

####### Material examined.

825 m: 13.XI.2015, LT, 4♂, 8♀. 851 m: 14.XI.2015, LT, 2♂, 5♀. 892 m: 16.X.2014, LT, 1♂, 1♀; 17.X.2014, LT, 1♀; 14.XI.2015, LT, 6♂, 13♀; 15.XI.2015, LT, 1♂, 3♀; 10.XII.2014, LT, 1♀. 1,325 m: 15.XI.2015, PT, 1♂.

####### General distribution and zoogeography.

OM, SA, YE. END_AR species.

####### Published records.

Paratypes known from the SANR, Baha (Häckel and Azadbakhsh 2016).

####### Remarks.

A frequent species that was collected during autumn from *Acacia* thorn woodlands with highest number of individuals collected by light traps at lower altitudes. Martin Häckel identified this species.

#### Paussinae

##### Paussini

###### Paussus cephalotes

Taxon classificationAnimaliaColeopteraCarabidae

Raffray, 1886

####### Material examined.

1562 m, 03.XI.2013, HP, 1♀.

####### General distribution and zoogeography.

SA, YE. END_AR species.

####### Published records.

Asir (Abdel-Dayem et al. 2018), Baha (El-Hawagry et al. 2013; Moore and Robertson 2014), Hejaz Mountains (Nagel 1982).

####### Remarks.

A rare species that was collected during autumn. The female of this species was found under a stone in a Barbary fig shrub community. Iftekhar Rasool identified this species and confirmed by Peter Nagel.

###### Paussus minutulus

Taxon classificationAnimaliaColeopteraCarabidae

Nagel & Rasool sp. n.

http://zoobank.org/EE392B1D-8B68-4CA1-A49C-61E4529093FD

Figs 4 , 5 , 6

####### Holotype (hereby designated).

Female; dry-mounted, glued on pinned pointed card; head with antennal clubs, left middle tibia with tarsus, left hind leg detached, and glued on to same card.

####### Original labels.

1. White, rectangular, black printed text and thin frame (*verbatim, slash = line break*): “**KSA.** Baha; / Shada Al-A’ Ala Nature Reserve; / 19°50.411'N, 41°18.686'E; / 1611 m; 2.IX.2015; PT.2; / Aldhafer, H., Fadl, H., Abdel-Dayem, M., / Elgharbawy, A., El Torkey, A., Soliman, A.”. 2. White, rectangular, black handwriting: “Paussus sp. 3”.

####### Added labels.

1. Red, rectangular, printed in black: “HOLOTYPUS/ *Paussusminutulus* / Nagel & Rasool, 2018”. 2. White label, printed: “♀”.

####### Holotype repository.

King Saud University Museum of Arthropods (KSMA), Department of Plant Protection, College of Food and Agriculture Sciences, King Saud University, Riyadh, Saudi Arabia.

####### Type locality.

Arabian Peninsula, western Saudi Arabia, Baha Province, Upper Shada Mountain, Shada Al-A’ Ala Nature Reserve 19°50.411'N, 41°18.686'E), 1611 m, pitfall trap no. 2, emptied on 2 September 2015 after 48 hours of exposure. The place of exposure of pitfall trap no. 2 is dominated by the cactus pear *Opuntiaficus*-*indica* (L.) Mill. (Cactaceae). This “cactus zone” covers the area of the nature reserve above approximately 1500 m. It is an extensively cultivated area, also characterized by small-scale terraced fields (Fig. 4). More details are given in “Study area” above.

**Figure 3.**
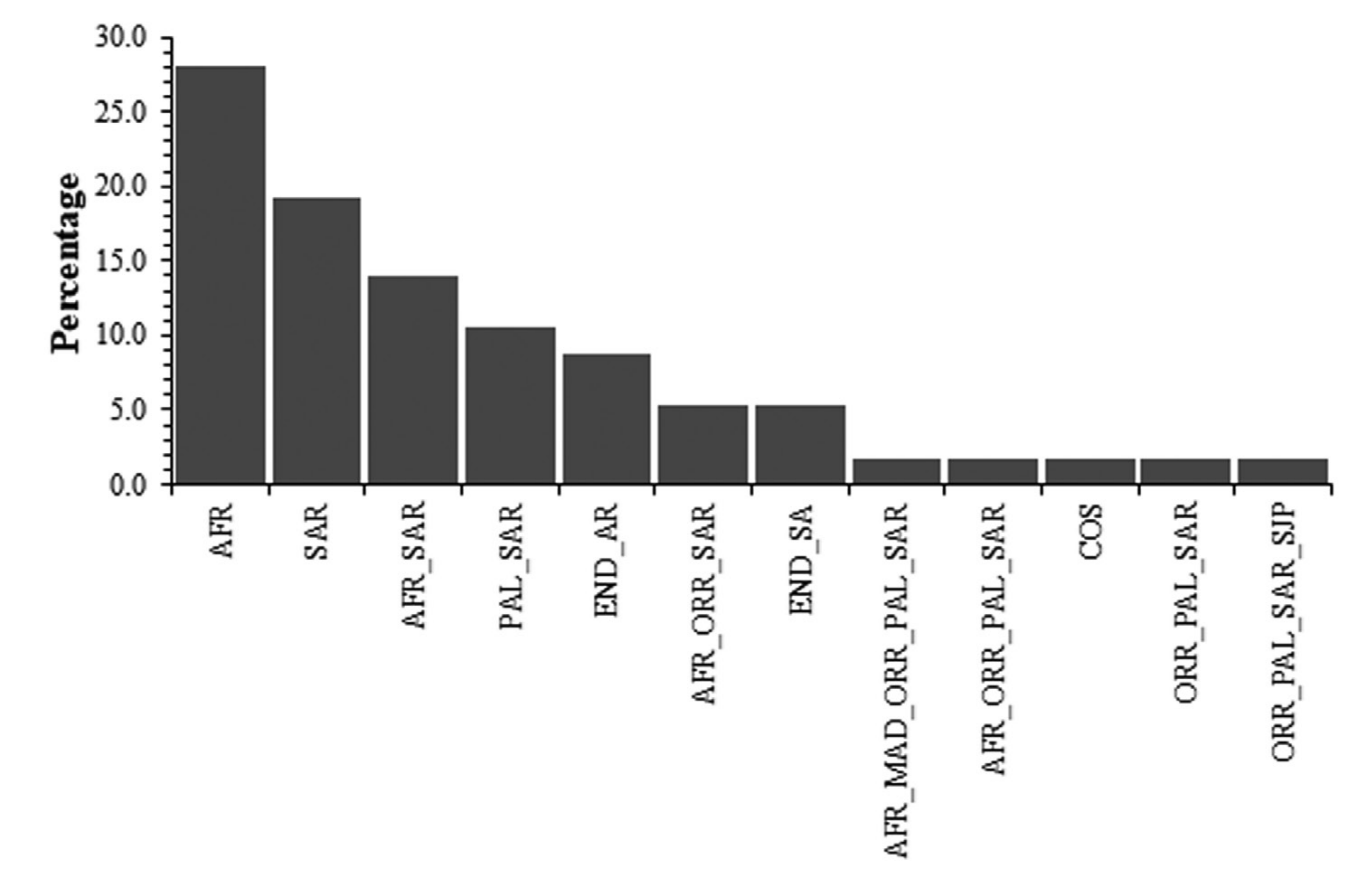
Zoogeographical affinities of the carabid fauna of Shada Al-A’Ala Nature Reserve, southwestern Saudi Arabia. AFR, Afrotropical; COS, Cosmopolitan; END_AR, endemic to the Arabian Peninsula; END_SA, endemic to Saudi Arabia; MAD, Madagascan; ORR, Oriental; PAL, Palaearctic; SAR, Saharo_Arabian; SJP, Sino_Japanese.

**Figure 4.**
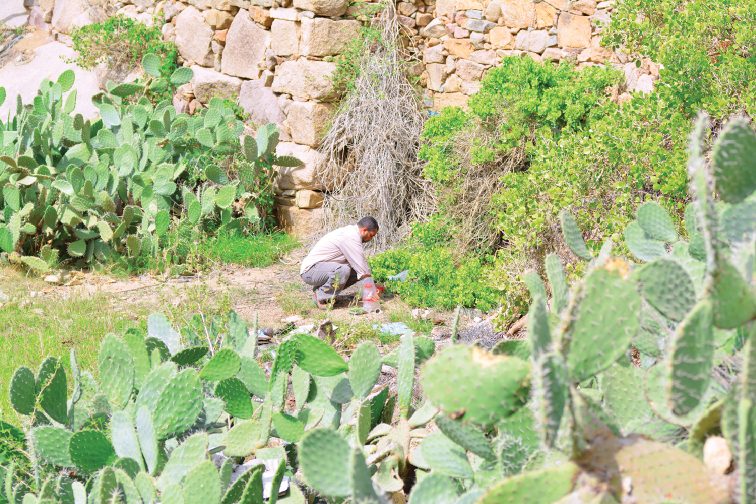
Photo of the cactus pear, *Opuntiaficus*-*indica* (L.) Mill. (Cactaceae) in the foreground and background, and the shrubs *Cappariscartilaginea* Decne. (Capparaceae) in the middle ground; the type locality for *Paussusminutulus* Nagel & Rasool, sp. n. at Shada Al-A’ Ala Nature Reserve, Baha Province, southwestern Saudi Arabia at an elevation of 1611 m.

####### Etymology.

The specific epithet is the Latin adjective for very small, because it is the smallest known Arabian *Paussus*.

####### Diagnosis.

*Paussusminutulus* sp.n. is a small *Paussus* of the *P.cucullatus* group *sensu lato* and is most similar to *P.abditus* Nagel, 2018 (SA) and *P.rougemontianus* Lorenz, 1998 (Yemen). It is distinguished from both by the tumid antennal club with its posterior basal angle large, thick, and apically truncate. Further specific characters are as follows: antennal club with excavation ending far in front of apex; head with vertex produced, with two distinct pores at the top; collar of anterior pronotum low, with transverse edge rounded and with lateral projections absent; pronotal trichome well developed at both ends of transverse furrow; pygidium with lower (posterior) margin with dense fringe of hair; fore and middle legs robust, not compressed, hind femur dilated and flattened, hind tibiae little wider than thick; small, apically fringed setae present at several body parts, most obvious at antennomere 1 and all legs.

####### Description of female holotype specimen.

**Figure 5.**
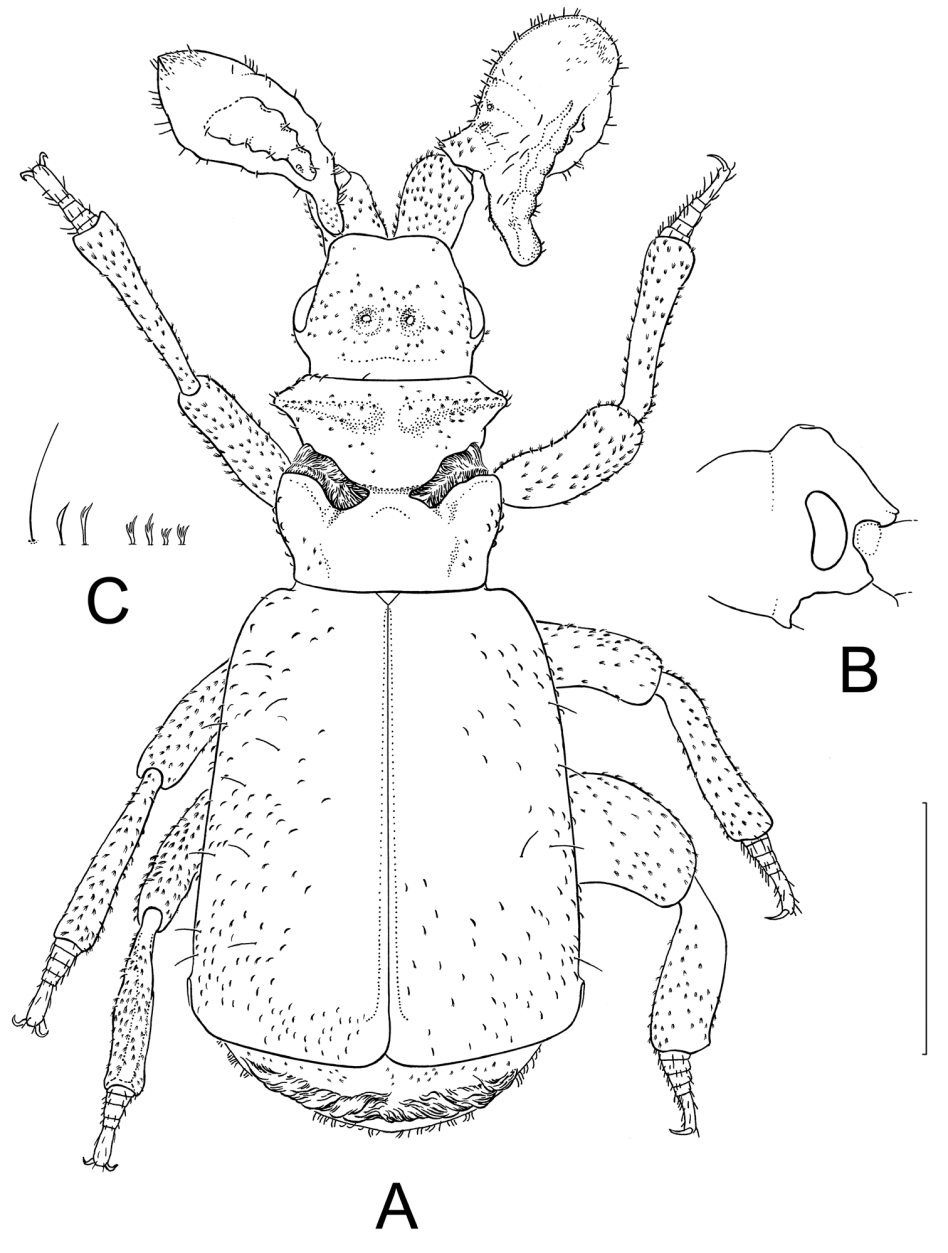
Paussus (Hylotorus) minutulus Nagel & Rasool, sp. n. **A** Habitus, dorsal view, appendices of right and left side at broadest and narrowest view respectively **B** Head, lateral view **C** Types of setae of dorsal pubescence, from left to right: one long, thin, hair-like seta; two shorter, lanceolate bristles, the right one with split apex; four short scaliform setae with multiply split, fringed apex; the setae are shown at enlarged view and at sizes relative to each other, yet without scale. Scale bar: 1 mm. Illustration: Adrian Gertsch. Copyright: Peter Nagel.

**Figure 6.**
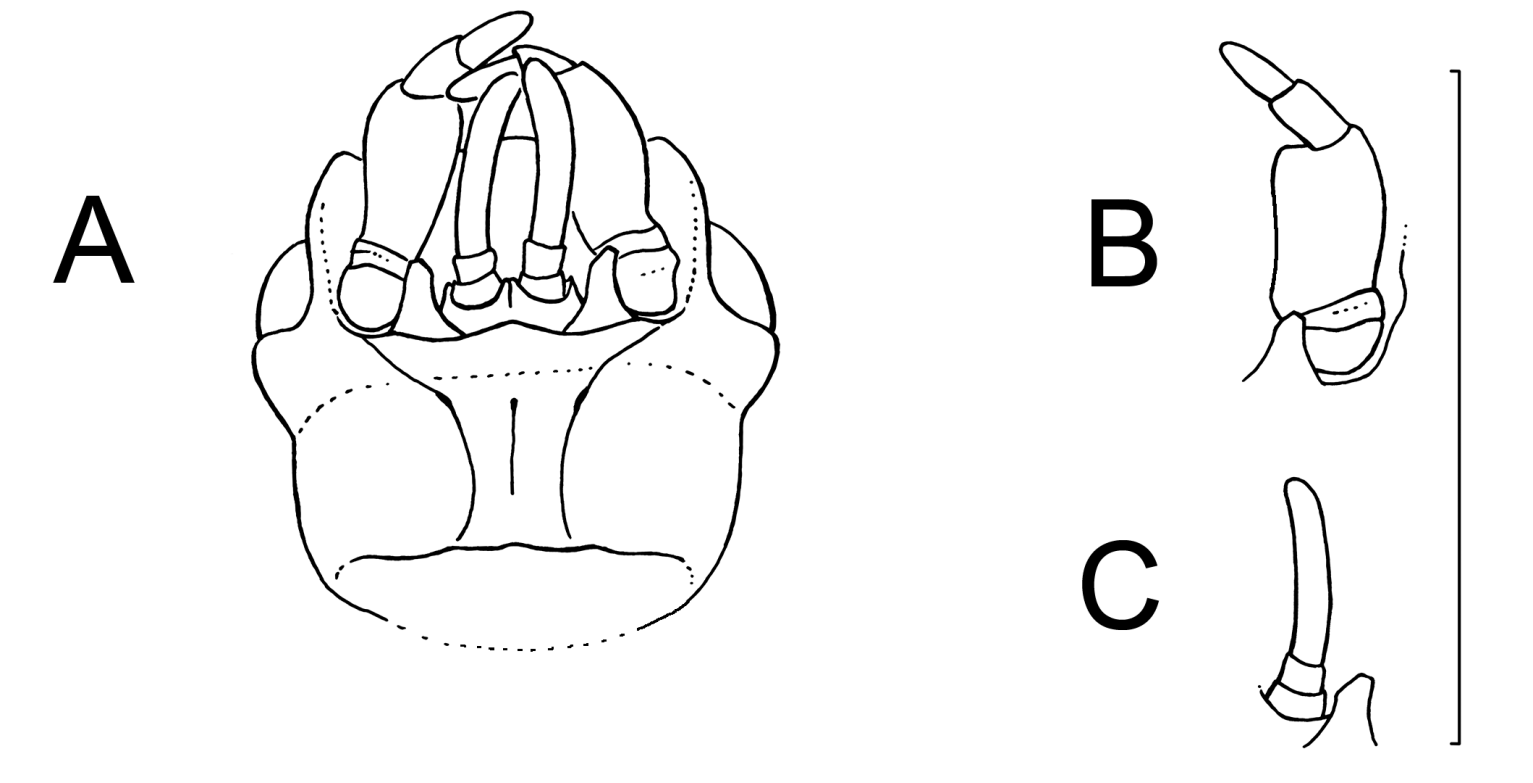
Paussus (Hylotorus) minutulus Nagel & Rasool, sp. n. **A** Head, ventral view **B** Left maxillary palpus, broadest view **C** Left labial palpus, broadest view. Scale bar: 1 mm. Illustration: Adrian Gertsch. Copyright: Peter Nagel.

(Fig. 5) Standardized body length from tip of head to tip of elytra 3.4 mm (3.5 mm total body length from tip of head to tip of pygidium), width across mid-elytra 1.5 mm. Body castaneous, appendices little darker, ventral abdomen and anterior part of pronotum little lighter; compacted or reinforced marginal areas of head, antennal clubs and pronotum narrowly blackish. Surface smooth and shining, except forehead matt and antennomere 1 (scape) with dense and coarse, yet shallow punctuation. Pubescence of elytra inconspicuous, restricted mostly to apical and lateral parts (abraded in the middle?), of short and upright as well as recumbent, narrowly lanceolate setae; elytra additionally with very few, long, thin, upright setae on lateral part of disc and as *series umbilicata*; head, pronotum, pygidium and appendices with scattered, short, mostly appressed setae; these short setae have a multiple split (fringed) apex and are most obvious at antennomeres 1, femora and tibiae, yet absent at elytra; antennal clubs with the normal apical sensory field and with scattered upright or slightly curved medium setae. Head 1.3 times wider than long, frontal margin broadly truncated, slightly biconvex; in dorsal view head in front of eyes little narrowed apically, gena and eye of equal length; temples not projecting; head with vertex produced, with two distinct pores at the top; pores broadly marginate with orifice slightly sunk. Antennomere 1 devoid of distinctly marked longitudinal edges; antennal club excavate on its exterior (posterior) side, 1.5 times longer than wide (basal tooth disregarded), tumid; frontal margin distinctly emarginate near base, with one small and one tiny fenestriform pit; anterior basal angle of club acute, marked; posterior basal projection large, thick, apically truncate; hind (exterior) side of the club with excavation limited by broad dorsal and ventral borders, and ending far before apex; at posterior view dorsal and ventral margins of excavation swollen, undulate with 3 or 4 low tubercles, each with one to three subapical setae; club without distinct trichome near ventral base, just an indistinct assemblage of three slightly thicker setae. Mouthparts as shown in Figure 6, not dissected; ligula at ventral view with longitudinal carinula in the middle of the disc (not shown in Fig. 6); (antepenultimate) maxillary palpomere II at broadest view 1.5 times as long as wide with mesal margin almost straight; (terminal) labial palpomere III long, narrow, five times as long as wide, apically rounded; gula with width/length ratio at narrowest point 0.9 (for measurement see Robertson and Moore (2016). Pronotum approximately as long as wide (1.1 times wider), transversely bipartite, with large trichomes at both ends of furrow; anterior part little wider than head (1.2 times), low, with transverse dorsal edge of collar broadly rounded, slightly indented in the middle, not angulate, lateral angles obscure; posterior part narrowed towards base. Elytral pubescence of two types: a few, very scattered, thin, long, erect setae on lateral parts of the disc (in addition to the similarly looking hairs of the *series umbilicornis*), plus scattered, recumbent, narrowly lanceolate setae on lateral and apical parts of disc; lateral subapical folds (“flange of Coanda”) normal, without peculiarities. Hind wings present. Pygidium with central disc almost even, with indistinct microsculpture, weakly shining; lower (anatomically posterior) margin explanate; pygidial trichome of dense fringe of long hair set semicircular along lower margin; ventral part of explanate margin set with one row of short, appressed setae. Legs robust, femora and tibiae of fore and middle legs not compressed, hind femur and tibia slightly compressed and dilated, the whole inner side of hind tibia with longitudinal shallow groove; pubescence of femora and tibiae of scattered, small, apically fringed setae; all tibiae without terminal spurs; terminal tarsomere of posterior tarsus as long as three preceding ones together; tarsomeres ventrally with few lateral setae, and glabrous in the middle. Inner side of hind femur subbasally with file of stridulatory organ; the file consists of multiple parallel fine grooves and is located at both the anterior and posterior parts of a longitudinal, short carinula.

####### Male.

Unknown.

####### Distribution.

The new species is only known from the holotype female specimen from the type locality at Shada Al-A’ Ala Nature Reserve.

####### Ecology.

This single specimen of *Paussus* was found in an area with low impact small-scale agriculture. The vegetation is characterized by dominant cactus pear. The altitude is ca 1610 m, the winters are cool and most of the 200 mm annual rainfall is concentrated between March and May (see details in chapter “Study area” above). The specimen of *P.minutulus* sp.n. was taken by a pitfall trap which also caught ants of the following taxa: *Camponotusaegyptiacus* Emery, 1915; *Messorebeninus* Santschi, 1927; *Monomoriumjizane* Collingwood & Agosti, 1996; *Pheidole* Westwood, 1839, sp.; *Tetramoriumsimillimum* Smith, 1851) and *Tetramoriumsericeiventre* Emery, 1877. Members of all these genera are known as host of one or more species of Paussini (Geiselhardt et al. 2007). *Paussusminutulus* sp.n. forms part of the Afrotropical *P.cucullatus* species group, of which several members are known to be associated with *Pheidole* sp., including the Arabian *P.rougemontianus* (see, for example, Luna de Carvalho 1989).

####### Remarks.

The new species is assigned to PaussussubgenusHylotorus Dalman, 1823, according to the phylogenetically based classification of Robertson and Moore (2016) (see Nagel et al. 2017a). The description of *P.minutulus* sp.n. given above agrees generally well the diagnosis and characters used in the key provided by Robertson and Moore (2016) and their subgenus description. The labial palpomere III is longer and narrower than described as usual for the Subgenus Hylotorus, yet still within the range of variation of this character, and similar to *P.abditus* Nagel, 2018 in Abdel-Dayem et al. (2018). The lacinia was not dissected. The fringed setae are conspicuous, despite their tiny size. Sometimes, individuals of a few *Paussus* species were found to show the same type of setae with both split and unsplit apices. In these cases, the splitting is most probably an artifact caused by a treatment during or after collecting (use of certain chemicals in the killing bottle or relaxing chamber, for example). In *P.minutulus* sp.n. this particular shape is obviously an intrinsic character, because they are alike at all body parts where they occur.

*Paussusminutulus* sp.n. forms part of the *P.cucullatus* group *sensu lato* and is most similar to and possibly part of the same clade as *P.abditus* Nagel, 2018 in Abdel-Dayem et al. (2018) (SA) and *P.rougemontianus* Lorenz, 1998 (Yemen) (replacement name for *Cochliopaussusrougemonti* Luna de Carvalho, 1989). It differs from *P.abditus* by the slightly shorter pronotum, the less compressed and dilated hind leg, the absence of a distinct subbasal antennal trichome, the smaller extension of the excavation at the posterior antennal club, the large, thick, apically truncated posterior basal projection of the antennal club, the presence of fringed setae, and the clearly marked, distinct cephalic orifices.

It differs from *P.rougemontianus* by the slightly shorter pronotum, the less compressed and dilated hind leg, the absence of a distinct subbasal antennal trichome, the dorsal hind margin of the antennal club retracted, the presence of fringed setae, and, above all, the large, thick, apically truncated posterior basal projection of the antennal club in the new species. The new species differs from the little-known Ethiopian *P.cyathiger* Raffray, 1886, among others, by the long, thin peg-like posterior basal angle of the antennal club, and the longitudinal crescent margin at the external part of the cephalic pores of the latter (see Abdel-Dayem et al. 2018).

It is the smallest known Arabian *Paussus* and at the same time it is among the smallest *Paussus* at global scale (smallest measurements 3.3–3.5 mm total body length): Paussus (Subg.incertaesedis) exiguus Reichensperger, 1929, Sudan; P. (Anapaussus) asperulus Fairmaire, 1898, Madagascar; P. (Anapaussus) pipitzi Dohrn, 1884 [ssp. pictor Reichensperger, 1922, and possibly further subspecies], Madagascar and P. (Edaphopaussus) favieri Fairmaire, 1851, southwestern Europe and northwestern North Africa).

The Arabian Peninsula and adjacent regions harbour ten (with *P.minutulus* sp.n. included) species of Paussinae, all members of the genus *Paussus* Linnaeus, 1775 (see Nagel et al. 2017b; Abdel-Dayem et al. 2018). The following eight species have been recorded from the montane ecoregion of southwestern SA and Yemen (linked to the Eastern Afromontane Highlands Hotspot, see Mittermeier et al. 2004). The presence of all of them is in line with the zoogeographical affiliation of southwestern Arabian Peninsula to the Afrotropical Region: *Paussusabditus* Nagel, 2018 (END_SA); *P.arabicus* Raffray, 1886 (AFR_SAR); *P.brittoni* Reichensperger, 1957 (END_YE); *P.cephalotes* Raffray, 1886 (END_AR); *P.cirenaicus* Fiori, 1914 (PAL_SAR); *P.minutulus* Nagel & Rasool, sp.n. (END_SA); *P.rougemontianus* Lorenz, 1998 (END_YE) and *P.thomsonii* Reiche, 1860 (PAL_AFR_SAR).

#### Pterostichinae

##### Abacetini

###### Abacetus crenulatus

Taxon classificationAnimaliaColeopteraCarabidae

Dejean, 1831

####### Material examined.

892 m: 23.IV.2014, LT, 2 exs; 1,225 m: 05.V.2015, LT, 1 ex. 1,325 m: 03.VI.2015, LT, 1 ex.

####### General distribution and zoogeography.

BF, BJ, CI, ML, MR, SA (Abdel-Dayem et al. 2018), SN, TD. AFR species.

####### Published records.

Asir (Abdel-Dayem et al. 2018). New provincial records for Baha.

####### Remarks.

A rare species collected during spring by light trapping in *Acacia* thorn woodlands. Mahmoud Abdel-Dayem identified this species.

###### Abacetus quadrisignatus

Taxon classificationAnimaliaColeopteraCarabidae

Chaudoir, 1876

####### Material examined.

471 m, 10.XII.2014, HP, 2 exs. 892 m, 15.II.214, LT, 2 exs.

####### General distribution and zoogeography.

ET, ER and YE (Socotra Island) (Felix et al. 2012). New country record. This range exemplifies AFR realm.

####### Remarks.

A rare species that was collected during autumn and winter from lower elevations in area of *Acacia* thorn woodlands. Mahmoud Abdel-Dayem identified this species.

#### Scaritinae

##### Clivinini

###### Coryza beccarii

Taxon classificationAnimaliaColeopteraCarabidae

Putzeys, 1873

####### Material examined.

471 m, 15.XI.2015, LT, 1 ex; 10.XII.2014, LT, 2 exs; 10.XII.2014, HP, 15 exs.

####### General distribution and zoogeography.

EG (Sinai), ER, GN, IL, OM, SA, YE. AFR_SAR species.

####### Published records.

Asir (Basilewsky 1979), Jizan and Makkah (Britton 1948, Balkenohl 1994). New provincial records for Baha.

####### Remarks.

A frequent species that was collected during autumn at lower altitudes. The adults were caught by hand picking under stones and by light trapping along the edge of a freshwater stream. Michael Balkenohl and Ali Elgharbawy identified this species.

##### Dyschiriini

###### Dyschirius chalybeus gibbifrons

Taxon classificationAnimaliaColeopteraCarabidae

Apfelbeck, 1899

####### Material examined.

825 m: 15.XI.2015, LT., 1 ex. 851 m: 15.XI.2015, LT., 1 ex. 892 m: 16.II.2014, LT, 1 ex; 23.IV.2014, LT, 1 ex; 15.XI.2015, LT, 2 exs. 1225 m, 17.X.2014, LT, 4 exs. 1,325 m: 02.IX.2015, LT, 2 exs; 17.X.2014, LT, 11 exs; 14.XI.2015, LT. 12 exs. 1,474 m: 15.II.2014, LT, 1 ex; 14.XI.2015, LT., 1 ex. PAL_SAR species.

####### General distribution and zoogeography.

AL, AT, AZ, BG, CZ, GE, GR, IL, IR, IT, MD, RO, RS, RU (South European Territory), SA (Balkenohl 1994), SK, TM, TR, UA. PAL_SAR species.

####### Published records.

Asir (Abdel-Dayem et al. 2018), Baha (Balkenohl 1994).

####### Remarks.

A frequent species that was recorded during all seasons with more individuals were collected during autumn. The adults were caught using light traps set in *Acacia* thorn woodlands. Michael Balkenohl, Ali Elgharbawy and Mahmoud Abdel-Dayem identified this species.

##### Scaritini

###### Scarites striatus

Taxon classificationAnimaliaColeopteraCarabidae

Dejean, 1825

####### Material examined.

1,225 m: 23.VIII.2014, PT, 3 exs. 1,325 m: 08.XII.2014, HP, 2 exs. 1,666 m: 02.IX.2015, PT, 1 ex; 15.XI.2015, HP, 5 exs.

####### General distribution and zoogeography.

DZ, EG (including Sinai), LY, SA, TN, YE. SAR species.

####### Published records.

Asir, Makkah (Balkenhol 1994; Abdel-Dayem et al. 2018). New provincial records for Baha.

####### Remarks.

A rare species that was collected during late summer and autumn from *Acacia* thorn woodlands and Barbary fig shrub communities. Michael Balkenohl, Ali Elgharbawy and Mahmoud Abdel-Dayem identified this species.

###### Scarites terricola aethiopicus

Taxon classificationAnimaliaColeopteraCarabidae

Bänninger, 1933

####### Material examined.

471 m: 15.XI.2015, LT, 1 ex; 10.XII.2014, HP, 1 ex.

####### General distribution and zoogeography.

DZ, EG (Sinai), ER, ET, IL, OM, SA, YE. AFR_SAR species.

####### Published records.

Baha, Eastern Province, Jizan, Madinah, Makkah, Riyadh (Britton 1948; Balkenohl 1994).

####### Remarks.

A rare species collected during autumn at lower altitudes in *Acacia* thorn woodlands. The adults were caught along the edge of a freshwater stream by hand picking under stones and by using light traps. Michael Balkenohl, Ali Elgharbawy and Mahmoud Abdel-Dayem identified this species.

#### Trechinae

##### Bembidiini

###### Bembidion atlanticum atlanticum

Taxon classificationAnimaliaColeopteraCarabidae

Wollaston, 1854

####### Material examined.

892 m, 16.II.2014, LT, 1♂; 17.X.2014, LT, 1♀. 1,225 m: 02.IX.2015, LT, 1♀. 1,325 m: 15.XI.2015, LT, 1♀. 1,611 m: 27.I.2015, LT, 3♂, 1♀.

####### General distribution and zoogeography.

AE, AF, AM, AZ, BG, CV, CY, DZ, EG (including Sinai), FR, GE, GR, IL, IN, IQ, IR, KG, KZ, MA, MD, MR, MT, NE, PT, RU (South European Territory), SA, SY, TD, TJ, TM, TN, TR, UA, UZ, YE. AFR_ORR_PAL_SAR species.

####### Published records.

Asir, Baha and Riyadh (Basilewsky 1979; El-Hawagry et al. 2013; Abdel-Dayem et al. 2018).

####### Remarks.

A rare species that was collected during late summer, autumn, and winter by light trapping in *Acacia* thorn woodlands and Barbary fig shrub communities. Paolo Neri and Mahmoud Abdel-Dayem identified this subspecies.

###### Bembidion niloticum niloticum

Taxon classificationAnimaliaColeopteraCarabidae

Dejean, 1831

####### Material examined.

825 m, 15.XI.2015, LT, 2♂, 3♀. 851 m: 15.XI.2015, LT, 2♂, 2♀. 892 m: 16.II.2014, LT, 2♂; 15.XI.2015, LT, 1♀. 1,225 m: 03.VI.2014, LT, 1♀; 14.XI.2015, LT, 1♂, 2♀.

####### General distribution and zoogeography.

AE, AF, AM, AZ, BG, CN, CY, EG, GE, GR, IL, IN, IQ, IR, JO, JP, KG, KH, KP, KR, KZ, MM, NP, OM, PH, PK, RU (South European Territory), SA, SY, TM, TR, TW, UZ, VN. ORR_PAL_SAR_SJP species.

####### Published records.

Asir (Abdel-Dayem et al. 2018), Makkah (Britton). New record for Baha Province.

####### Remarks.

A frequent species that was sporadically collected during autumn, winter, and spring in *Acacia* thorn woodlands. Paolo Neri and Mahmoud Abdel-Dayem identified this subspecies.

###### Sphaerotachys conspicuus

Taxon classificationAnimaliaColeopteraCarabidae

(Schaum, 1863)

####### Material examined.

471 m: 2.III.2015, LT, 4 exs. 825 m, 15.XI.2015, LT, 15 exs. 851 m, 14.XI.2015, LT, 3 exs; 15.XI.2015, LT, 17 exs. 892 m: 23.IV.2014, LT, 1 ex. 1,225 m: 2.III.2015, LT, 41 exs; 05.V.2015, LT, 1 ex; 03.VI.2014, LT, 1 ex; 2.IX.2015, LT, 2 exs; 14.XI.2015, LT, 4 exs. 1,325 m: 27.I.2015, LT, 27 exs; 15.II.2014, LT, 1 ex; 2.III.2015, LT, 108 exs; 21.IV.2014, LT, 1 ex; 05.V.2015, LT, 1 ex; 03.VI.2014, LT, 6 exs; 17.X.2014, LT, 2 exs; 15.XI.2015, LT, 5 exs. 1,474 m: 15.II.2014, LT, 1 ex; 2.III.2015, LT, 7 exs; 05.V.2015, LT, 1 ex. 1,563 m: 27.I.2015, LT, 1 ex; 2.III.2015, LT, 4 exs; 05.V.2015, LT, 2 exs. 1,611 m: 27.I.2015, LT, 2 exs; 2.III.2015, LT, 1 ex; 05.V.2015, LT, 1 ex. 1614 m, 20.X.2014, LT, 3 exs. 1,666 m: 27.I.2015, LT, 1 ex; 05.V.2015, LT, 1 ex.

####### General distribution and zoogeography.

AE, AO, DZ, EG, ER, ET, IL, KE, LY, MR, NE, SA, SD, SO, TD, YE. AFR_SAR species.

####### Published records.

Asir (Basilewsky 1979; Abdel-Dayem et al. 2018), Baha (El-Hawagry et al. 2013).

####### Remarks.

An abundant species, which was collected during all four seasons, with highest number of individuals collected during winter (March) and lowest numbers during the late summer (September). The adults were caught by light traps set at various altitudinal zones (471–1666 m) in *Acacia* thorn woodlands and Barbary fig shrub communities. Mahmoud Abdel-Dayem identified this species.

###### Sphaerotachys tetraspilus variabilis

Taxon classificationAnimaliaColeopteraCarabidae

(Chaudoir, 1876)

####### Material examined.

471 m: 03.III.2015, LT, 11 exs. 851 m, 15.XI.2015, LT, 1 ex. 892 m: 23.IV.2014, LT, 2 exs. 1,325 m: 27.I.2015, LT, 53 exs. 15.II.2015, LT, 1 ex. 17.X.2014, LT, 1 ex. 1,474 m: 26.I.2015, PT, 5 exs. 27.I.2015, LT, 57 exs; 15.II.2014, LT, 2 exs; 02.III.2015, PT, 1 ex; 05.V.2015, LT, 5 exs; 18.X.2014, PT, 1 ex. 1,563 m: 26.I.2015, PT, 2 exs; 27.I.2015, LT, 7 exs; 02.III.2015, LT, 8 exs; 05.V.2015, LT, 3 exs. 1,611 m: 27.I.2015, LT, 25 exs; 02.III.2015, LT, 5 exs; 05.V.2015, LT, 25 exs; 03.VI.2014, LT, 1 ex; 17.X.2014, LT, 1 ex. 1,666 m: 27.I.2015, LT, 1 ex; 02.III.2015, LT, 1 ex; 05.V.2015, LT, 5 exs.

####### General distribution and zoogeography.

AE, AO, BF, CD, CI, CV, DZ, ER, ET, GM, IN, KE, ML, MR, NE, PK, SA, SD, SN, SO, TD, UG. AFR_ORR_SAR species.

####### Published records.

Asir (Basilewsky 1979; Abdel-Dayem et al. 2018), Baha (El-Hawagry et al. 2013), Makkah (Britton 1948).

####### Remarks.

A common species that was collected during autumn, winter, and spring, with a peak during winter. The adults were collected at various altitudinal zones in *Acacia* thorn woodlands and Barbary fig communities. Mahmoud Abdel-Dayem identified this subspecies.

###### Tachyura biblis

Taxon classificationAnimaliaColeopteraCarabidae

(Britton, 1948)

####### Material examined.

471 m: 03.III.2015, LT, 3 exs. 963 m: 03.XI.2013, HP, 1 ex. 1225 m, 02.III.2015, LT, 6 exs; 05.V.2015, LT, 2 exs. 1,325 m: 27.I.2015, LT, 2 exs; 02.III.2015, LT, 24 exs; 21.IV.2014, LT, 2 exs; 05.V.2015, LT, 19 exs; 23.VIII.2014, LT, 1 ex. 1,474 m: 27.I.2015, LT, 1 ex; 02.III.2015, LT, 14 exs; 05.V.2015, LT, 8 exs. 1,563 m: 02.III.2015, LT, 3 exs; 05.V.2015, LT, 6 exs. 1,611 m: 02.III.2015, LT, 2 exs; 21.IV.2014, LT, 1 ex; 05.V.2015, LT, 3 exs; 03.VI.2014, SW, 1 ex; 27.VII.2015, LT, 1 ex. 1,666 m: 05.V.2015, LT, 1 ex; 27.VII.2015, LT, 1 ex; 17.X.2014, LT, 1 ex.

####### General distribution and zoogeography.

AE, DJ, DZ, IR, MR, NE, SA, TD, YE. AFR_SAR species.

####### Published records.

Asir (Abdel-Dayem et al. 2018), Riyadh (Basilewsky 1979).

####### Remarks.

A common species, which was collected during all seasons of the year with the peak reached during winter (March). The adults were collected from both major plant communities in the SANR and from a wide altitudinal range (471–1666 m). Mahmoud Abdel-Dayem identified this species.

##### Trechini

###### Perileptus areolatus

Taxon classificationAnimaliaColeopteraCarabidae

Creutzer, 1799

####### Material examined.

1,225 m: 27.I.2015, LT, 2 exs; 02.III.2015, LT, 11 exs; 21.IV.2014, LT, 1 ex. 1,325 m: 27.I.2015, LT, 1 ex; 02.III.2015, LT, 2 exs; 05.V.2015, 2 exs. 1,474 m: 27.I.2015, LT, 2 exs; 02.III.2015, LT, 2 exs. 1,563 m: 21.IV.2014, LT, 1 ex; 05.V.2015, LT, 1 ex. 1,611 m: 27.I.2015, LT, 1 ex. 1,666 m: 03.V.2015, PT, 1 ex.

####### General distribution and zoogeography.

AL, AM, AT, AZ, BA, BE, BG, CH, CZ, DE, DZ, ES, FR, GB, GE, GR, HR, HU, IE, IL, IR, IT, LT, LV, MA, MD, MK, NO, PL, PT, RO, RU, SA, SE, SI, SK, SY, TN, TR, UA. PAL_SAR species.

####### Published records.

Makkah (Britton 1948). New record for Baha Province.

####### Remarks.

A frequent species that was collected during winter and spring from *Acacia* thorn woodlands and Barbary fig communities. Mahmoud Abdel-Dayem identified this species.

###### Perileptus rutilus

Taxon classificationAnimaliaColeopteraCarabidae

Schaum, 1860

####### Material examined.

471 m: 03.III.2015, LT, 2 exs; 10.XII.2014, HP, 1 ex; 10.XII.2014, LT, 1 ex. 1,325 m: 02.III.2015, LT, 2 exs; 05.V.2015, LT, 2 exs. 1,474 m: 02.III.2015, PT, 1 ex; 02.IX.2015, LT, 1 ex.

####### General distribution and zoogeography.

EG, SA (Abdel-Dayem et al. 2018), SD, TD, YE. SAR species.

####### Published records.

Asir (Abdel-Dayem et al. 2018). New provincial record for Baha.

####### Remarks.

A rare species that was collected during all seasons from *Acacia* thorn woodlands. Mahmoud Abdel-Dayem identified this species.

###### Perileptus testaceus

Taxon classificationAnimaliaColeopteraCarabidae

Putzeys, 1870

####### Material examined.

825 m: 15.XI.2015, LT, 3 exs. 851 m: 15.XI.2015, LT, 7 exs. 1,225 m: 14.XI.2015, LT, 1 ex. 1,325 m: 14.XI.2015, LT, 1 ex; 15.XI.2015, LT, 1 ex. 1,474 m: 05.V.2015, LT, 1 ex. 1,563 m: 21.IV.2014, LT, 1 ex.

####### General distribution and zoogeography.

AE, DJ, ET, OM, SA (Abdel-Dayem et al. 2018), SO, YE. AFR species.

####### Published records.

Asir (Abdel-Dayem et al. 2018). New provincial record for Baha.

####### Remarks.

A frequent species that was collected during spring and autumn by light trapping in *Acacia* thorn woodlands and Barbary fig communities. Mahmoud Abdel-Dayem identified this species.

## Discussion

The Carabidae of SA, comprising the ground and tiger beetles, has been reviewed with currently approximately 183 recognized species (Abdel-Dayem et al. 2018). However, not every SA province has been equally surveyed and studied. The highlands of the southwestern SA are a major hotspot of biodiversity (Hegazy et al. 1998; Heneidy and Bidak 2001), yet, the knowledge of the carabid diversity in this region is incompletely understood. This includes Baha Province, which includes SANR. Thirty species have been documented from Baha Province (Mateu 1986; Balkenohl 1994; El-Hawagry et al. 2013; Moore and Robertson; 2014; Häckel and Azadbakhsh 2016; Rasool et al. 2017, 2018a, b). The study of El-Hawagry et al. (2013) included the first recorded carabid species in the SANR, reporting only *Paussuscephalotes*. Häckel and Azadbakhsh (2016) and Rasool et al. (2018) documented 13 species in which *Lebiaraeesae* and *Microcosmodesarabicus* were newly described. It should be noted that none of these three studies specifically targeted this family for the SANR.

This study represents the first baseline inventory of the carabid beetles in SNAR, within the mountains in the southwestern Saudi Arabia. The study revealed 62 species belonging to 39 genera, 17 tribes and 10 subfamilies. This number of species represent about 33% of the total known carabid fauna of SA. Also, our study includes a new species, three species endemic to SA, six confined to Arabian Peninsula, four new country records, and 24 species recorded for the first time from Baha Province. The result expands the number of carabid species recorded from Baha to 67. The number of species in this current list is similar to that of Garf Raydah Nature Reserve (GRNR) in Asir Province (61 species), a much smaller area as compared to SANR (Abdel-Dayem et al. 2018). This may be due to the wide altitudinal range (1,150–2,820 m), high annual rainfall range (600–800 mm/annum), cool temperatures, relatively high humidity, and the presence of the last remnants of dense African pencil cedar forest, *Juniperusprocera* Hochst. ex Endl. (Cupressaceae) in GRNR (El-Juhany 2015, SWA 2018). Both nature reserves sharing about 64.5% (40 species) of the recorded carabid species. The Lebiini species are prevailed the carabid fauna of SNAR (30.6% of the total species), a similar finding was recently being reported from GRNR (Abdel-Dayem et al. 2018).

Biogeographically, SA is heterogeneous region that hosts an interesting mixture of biodiversity from Afrotropical, Palaearctic, and traces of Oriental realms due to its position between Africa and Eurasia (Büttiker 1979; Larsen 1984; Hölzel 1998). This mixture of taxa is also apparent in Baha Province, including SANR (El-Hawagry et al. 2016, 2017). The carabid fauna of the SANR is characterized by the prevalence of Afrotropical (28.1%) and Saharo-Arabian (19.3%) elements. The influence of the Palaearctic species is moderate (10.5%) and Oriental species is noticeably smaller (3.5%) (Fig. 3). Based on the zoogeographical analysis of the insect fauna, El Hawagry et al. (2013) suggested that the fauna of Baha Province is biologically related to the Afrotropical region rather than to the Palaearctic or Eremic zone and has little Oriental affinity. The specificity of the SANR carabid fauna is enhanced by a small fraction of endemics (5.3%). This percentage of endemic species is low compared to the percentage endemic species of the carabid fauna in Garf Raydah Nature Reserve (Abdel-Dayem et al. 2018).

In conclusion, our study provides a first account of the carabid beetle fauna of the SANR, Baha Province, in the southwestern SA. The SNAR has a relatively diverse carabid fauna (62 species), reflecting its rich flora. In its composition, the carabid fauna of SANR has almost the same number of species as GRNR, in Asir Province (Abdel-Dayem et al. 2018), and shares with the GRNR 64.5% of its species. The SNAR carabid fauna is mostly of Afrotropical origin with high influence of Saharo–Arabian and relatively little influence of the Oriental elements. The carabid fauna of the SNAR has a low level of endemism and high number of Lebiini species. Extensive surveying of the highlands in southwestern SA, may reveal further species. Beyond enhancing our knowledge of the SA carabid fauna, these results will provide useful information for guiding the conservation activities (Koivula 2011; Kotze et al. 2011) in the SANR and starting point to the future more detailed investigation on the fauna in the southwestern SA.

## Supplementary Material

XML Treatment for Brachinus crepitans

XML Treatment for Brachinus dorsalis

XML Treatment for Pheropsophus africanus

XML Treatment for Calosoma imbricatum

XML Treatment for Calosoma olivieri

XML Treatment for Calosoma senegalense

XML Treatment for Calomera alboguttata

XML Treatment for Cylindera rectangularis

XML Treatment for Myriochila melancholica

XML Treatment for Myriochila nudopectoralis

XML Treatment for Chlaenius canariensis seminitidus

XML Treatment for Chlaenius flavipes

XML Treatment for Chlaenius laeviplaga saudiarabica

XML Treatment for Chlaenius pachys

XML Treatment for Tetragonoderus arcuatus

XML Treatment for Tetragonoderus quadrum

XML Treatment for Amblystomus orpheus

XML Treatment for Amblystomus

XML Treatment for Anthracus angusticollis

XML Treatment for Crasodactylus punctatus

XML Treatment for Harpalus impressus

XML Treatment for Harpalus tenebrosus tenebrosus

XML Treatment for Progonochaetus planicollis

XML Treatment for Siopelus quadraticollis

XML Treatment for Apristus arabicus

XML Treatment for Calodromius mayeti

XML Treatment for Dromius buettikeri

XML Treatment for Eremolestes sulcatus

XML Treatment for Lebia auberti

XML Treatment for Lebia nilotica

XML Treatment for Lebia raeesae

XML Treatment for Matabele arabica

XML Treatment for Merizomena buettikeri

XML Treatment for Metadromius arabicus

XML Treatment for Metadromius brittoni

XML Treatment for Metadromius

XML Treatment for Microlestes discoidalis

XML Treatment for Microlestes infuscatus fragilis

XML Treatment for Pseudomesolestes quadriguttatus

XML Treatment for Singilis discoidalis

XML Treatment for Singilis

XML Treatment for Syntomus submaculatus

XML Treatment for Zolotarevskyella rhytidera

XML Treatment for Perigona nigriceps

XML Treatment for Cymbionotum microphthalmum

XML Treatment for Microcosmodes arabicus

XML Treatment for Paussus cephalotes

XML Treatment for Paussus minutulus

XML Treatment for Abacetus crenulatus

XML Treatment for Abacetus quadrisignatus

XML Treatment for Coryza beccarii

XML Treatment for Dyschirius chalybeus gibbifrons

XML Treatment for Scarites striatus

XML Treatment for Scarites terricola aethiopicus

XML Treatment for Bembidion atlanticum atlanticum

XML Treatment for Bembidion niloticum niloticum

XML Treatment for Sphaerotachys conspicuus

XML Treatment for Sphaerotachys tetraspilus variabilis

XML Treatment for Tachyura biblis

XML Treatment for Perileptus areolatus

XML Treatment for Perileptus rutilus

XML Treatment for Perileptus testaceus
